# Physiological performance of native and invasive crayfish species in a changing environment: insights from Dynamic Energy Budget models

**DOI:** 10.1093/conphys/coac031

**Published:** 2022-05-31

**Authors:** Nina Marn, Sandra Hudina, Ines Haberle, Ana Dobrović, Tin Klanjšček

**Affiliations:** 1 Division for Marine and Environmental Research, Rudjer Boskovic Institute, 10002 Zagreb, Croatia; 2 School of Biological Sciences, The University of Western Australia, Crawley, Western Australia 6009, Australia; 3 Department of Biology, Faculty of Science, University of Zagreb, 10000 Zagreb, Croatia

**Keywords:** Decapoda, invasion potential, life history traits, ontogeny, standard DEB model freshwater crayfish

## Abstract

Crayfish are keystone species important for maintaining healthy freshwater ecosystems. Crayfish species native to Europe, such as *Astacus astacus* and *Austropotamobius torrentium*, are facing decline and are increasingly endangered by changing climate and invasions of non-native crayfish, such as *Pacifastacus leniusculus* and *Procambarus virginalis*. The success of these invasions largely depends on differences in ontogeny between the native species and the invaders and how changes in the environment will affect the ontogeny. Dynamic Energy Budget (DEB) models can be used to investigate such differences because the models capture dependence of metabolism, and therefore ontogeny, on environmental conditions. We develop DEB models for all four species and investigate key elements of ontogeny and metabolism affecting interspecific competition. We then use the DEB models to predict individual growth and reproduction in current and new conditions that are expected to arise from climate change. Although observations suggest that *P. leniusculus* poses the major threat to native species, our analysis identifies *P. virginalis*, in spite of its smaller size, as the superior competitor by a large margin—at least when considering metabolism and ontogeny. Our simulations show that climate change is set to increase the competitive edge of *P. virginalis* even further. Given the prospects of *P. virginalis* dominance, especially when considering that it is able to withstand and spread at least some crayfish plague strains that severely affect native species, additional research into *P. virginalis* is necessary.

## Teaser

Crayfish help maintain healthy freshwater ecosystems but, if translocated, may take the detrimental role of an invasive species. Here, we used modeled energy acquisition and utilization of individuals to infer outcomes of competition between native and invasive crayfish based on their physiology, and the expected impacts of climate change.

## 1 Introduction

Freshwater crayfish belong to a diverse infraorder of decapod crustaceans (Astacidea) with nearly 700 known species distributed worldwide (Crandall & De Grave, 2017). Crayfish are keystone species that directly affect ecosystem processes and structure as well as species diversity (Pyšek & Richardson, 2010). They are among the largest benthic invertebrates that often dominate benthic biomass and have a relatively long life span, omnivorous feeding habits and are highly aggressive (Reynolds *et al.*, 2013; Usio & Townsend, 2008). Also, due to their high activity, which includes bioturbation and burrowing (Matsuzaki *et al.*, 2009; Statzner *et al.*, 2003), crayfish are considered to be ecosystem engineers. They have an intermediate trophic position and serve both as predators of other invertebrates and embryonic stages of vertebrates and as prey for a number of fish species and terrestrial vertebrates (Ilhéu *et al.*, 2007; Taylor *et al.*, 2019). Crayfish are also economically important and are cultivated in aquaculture both for food and for ornamental purposes (Holdich, 2002).

In the wild, populations of native crayfish are increasingly endangered and declining (Holdich *et al.*, 2009; Lodge *et al.*, 2000; Richman *et al.*, 2015) due to multiple anthropogenic pressures such as climate change, habitat fragmentation and introductions of non-indigenous crayfish species. Some crayfish species are especially successful invaders: they are among the most frequently introduced aquatic invertebrates with high number of documented negative impacts on native crayfish, freshwater biodiversity and ecosystem functioning and structure (Twardochleb *et al.*, 2013). Introduced invaders compete with native species for resources and often carry novel pathogens to which the native populations are very susceptible. As the pressures are expected to increase, successful conservation urgently requires better understanding of the mechanisms by which different pressures contribute to extinction risks for the native crayfish populations (Taylor *et al.*, 2019). At the same time, elucidating factors that drive crayfish establishment, population growth and dispersal is key to managing invasive crayfish species and could help identify stressors that may impede invasions.

Invasion success is influenced by a number of environmental variables, such as the similarity of the recipient community to that in the species’ native range and frequency of introduction events (Hayes & Barry, 2008; Kolar & Lodge, 2001), or species life history traits (e.g. growth rate, fecundity or tolerance to disturbance) that depend on invaders’ physiological characteristics (Kelley, 2014; Van Kleunen *et al.*, 2010). The ability to maintain fitness with respect to changing biotic (i.e., competition, predation) and abiotic (i.e., temperature, pollution) conditions is equally important for native and invasive species, but invaders usually exhibit higher plasticity in physiological tolerance (McCann *et al.*, 2018). Metabolic processes are among key factors that set the limits to physiological tolerance and will be affected by the intensity of environmental stress (Kelley, 2014).

We explore these issues using Dynamic Energy Budget (DEB) models, a proven tool linking stressors to their effects on metabolism and ontogeny of individuals (e.g., Baas *et al.*, 2010; Kooijman, 2018; Marn *et al.*, 2020; see also Lavaud *et al.*, 2021 and DEB library -DEB library of scientific publications containing 1043 items on 31 January 2022, with at least 32 of them containing ‘stress’ in the title: https://www.zotero.org/groups/500643/deb_library/library- for more examples. DEB models predict growth and reproduction of individuals as a function of environmental conditions. These predictions can then be up-scaled to predict population-level impacts of stressors. Indeed, DEB has already been recognized and proposed as a valuable tool in crayfish management and conservation (Taylor *et al.*, 2019) but, until recently, it has not been studiously applied to study freshwater crayfish species.

We started by developing DEB models for (i) two vulnerable and endangered native European species (*Astacus astacus*, the noble crayfish; *Austropotamobius torrentium*, the stone crayfish) and (ii) two successful invasive species (*Pacifastacus leniusculus*, the signal crayfish; *Procambarus virginalis*, the marbled crayfish). Models for *A. torrentium* and *P. virginalis* were newly developed, whereas models for *A. astacus* and *P. leniusculus* were expanded from the Add-my-Pet database of DEB models and parameters (AmP, 2021, Archived versions) by adding additional datasets derived from the literature and our own research.

We then used the models to explore differences in metabolic response to a range of different environmental conditions between native and invasive species. Food and temperature were chosen as the two most important factors that shape metabolic response, ontogeny and life history traits (Kouba *et al.*, 2010; Marn *et al.*, 2017a). Food availability was taken as an indirect measure of density-dependent effects: although food is not a limited resource in nature due to omnivory of crayfish, in populations of high density or when faced with a dominant competitor (invasive crayfish), feeding may be limited since individuals spend more time in competitive interactions than feeding and dominant individuals or species may hold the priority of access to optimal food sources (Hudina *et al.*, 2011a; Kubec *et al.*, 2019; Tricarico & Aquiloni, 2016). Temperature is a key abiotic factor in determining species distribution because temperature greatly affects cellular and biochemical processes (Johnston & Bennett, 2008; Somero, 2002; 2005), thus driving organismal performance (Pörtner, 2002). Accounting for effects of temperature on ontogeny may therefore shed light on invasion patterns of introduced species and help identify critical issues driving extinction of native species. Ectotherms such as crayfish are particularly sensitive because the lack of internal temperature control makes them highly dependent on environmental temperature. Deep understanding of effects of temperature is especially important given rapid climate-change driven environmental changes that may affect future invasion patterns and may accelerate population loss of endangered native crayfish species (Hudina *et al.*, 2011a; Lovrenčić *et al.*, 2022).

## 2 Methods

Here we describe models for the two native and two invasive crayfish species considered in this paper, outline the basics of DEB theory with a focus on the two main environmental factors affecting ontogeny (food and temperature) and describe additional simulations performed to elucidate the effects of food availability and temperature on selected life history traits of two direct competitors—the native *A. astacus* and invasive *P. leniusculus*.

### 2.1 Model organisms

The two native European crayfish species, *A. astacus* and *A. torrentium*, are considered vulnerable and endangered throughout Europe and are protected by national and EU regulations (EU Council Directive 92/43/EEC 1992). The conservation status of both species is assessed as unfavorable in all biogeographical regions of its occurrence and is deteriorating in almost all regions (Article 17 Web Tool, 2022). Chief causes of such declines include hydromorphological changes and habitat loss, pollution, introduction of (novel) diseases and displacement by invasive crayfish species (Article 17 Web Tool, 2022).

The first of the two invasive crayfish species considered in the study, *P. leniusculus*, is the most successful crayfish invader of European freshwaters (Kouba *et al.*, 2014); the second species, *P. virginalis*, is the emerging invader and the only known obligatory parthenogenetic freshwater crayfish species (Andriantsoa *et al.*, 2019; Kouba *et al.*, 2021; Vogt, 2020). Both invaders are potential vectors of crayfish plague, a disease caused by the oomycete pathogen *Aphanomyces astaci*, which is mostly lethal to native crayfish (Martín-Torrijos *et al.*, 2019). Transmission of the crayfish plague from tolerant invasive crayfish such as *P. leniucuslus* to susceptible native species such as *A. astacus* and *A. torrentium* is considered to be the major factor responsible for the decline of native populations throughout Europe (Martín-Torrijos *et al.*, 2019). However, even in the absence of crayfish plague agent, invasive crayfish frequently outcompete native species due to their advantageous life history traits (Dragičević *et al.*, 2020; Westman *et al.*, 2002).

While the native and invasive species analysed here reach similar size (i.e. *P. leniusculus* and *A. astacus* grow to similar size, as do *P. virginalis* and *A. torrentuim*; Table 3), both invaders mature earlier and have much higher fecundity compared with the native species (Table 3; Hossain *et al.*, 2018). Also, both invaders have wider optimal thermal niches, compared with the analysed native crayfish (Jaklič *et al.*, 2014; Vesely *et al.*, 2015; Vogt, 2020).

Finally, three of the studied species (*P. leniusculus*, *A. astacus* and *A. torrentium*) are closely phylogenetically related (family Astacidae) and have the following: (i) relatively similar habitat and food preferences; and (ii) synchronous life cycles characterized by mating in autumn, long egg incubation period that includes winter diapause and one offspring generation per year (Crandall & De Grave, 2017; Westman & Savolainen, 2002). By contrast, *P. virginalis*, is phylogenetically more distant (family Cambaridae; Vogt, 2020) and is characterized by an overall faster life cycle with much shorter life span, earlier maturation, shorter egg incubation period and subsequent multiple generations of offspring per year as a result of its parthenogenetic mode of reproduction (Vogt, 2020).

### 2.2 DEB model of crayfish

**Fig. 1:**
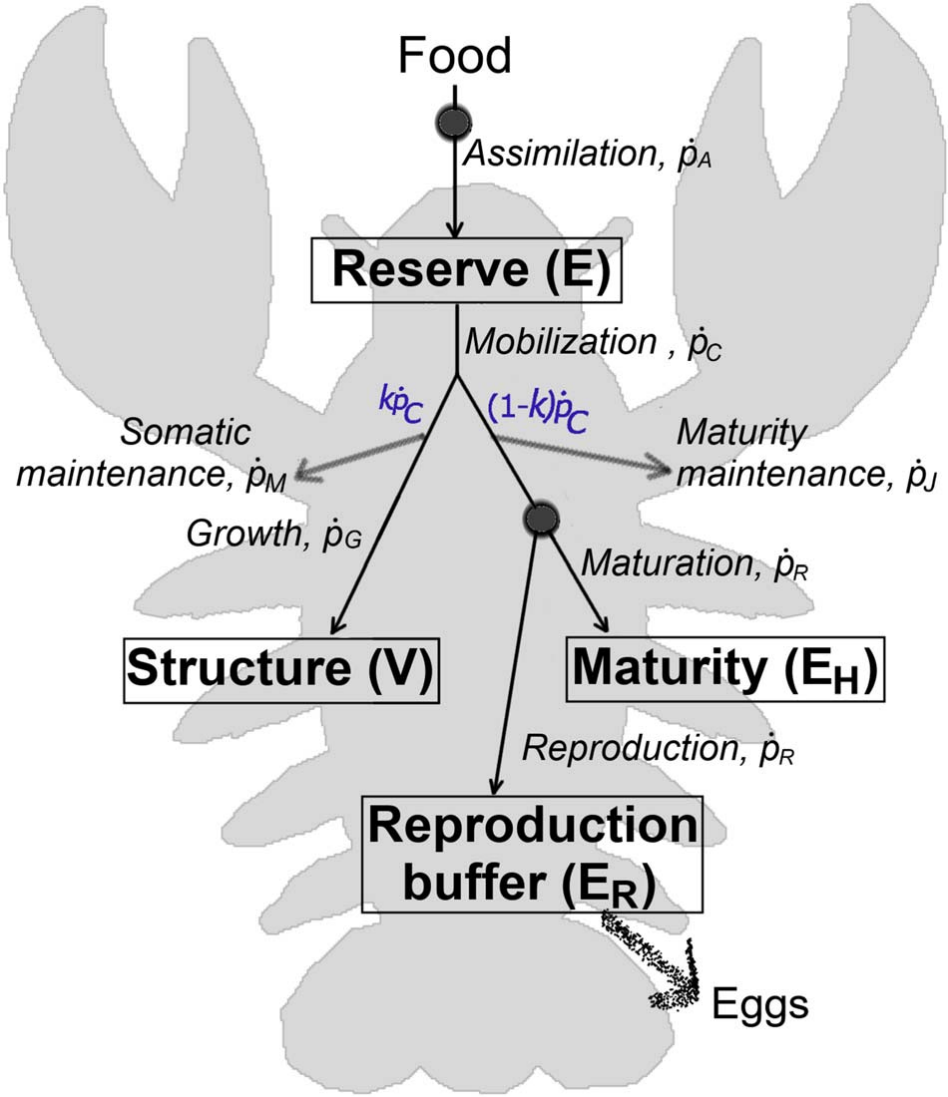
Conceptual representation of the metabolic processes. Solid arrows represent standard energy fluxes, and boxes mark state variables. Energy is assimilated from food into the reserve and subsequently allocated to fuel the metabolic processes: a fixed fraction $\kappa $ of the mobilized flux is allocated to somatic maintenance and growth, and the remaining fraction ($1-\kappa $) to increase and maintenance of maturity, or towards reproduction. Circles mark switches in energy pathways linked to transitions between life stages: (1) onset of feeding occurs at birth, when the individual transitions from embryo to juvenile stage; and (2) onset of investment into reproductions occurs at puberty, marking the transition from juvenile to adult life stage. For dynamics of state variables, the reader is referred to Table 1.

DEB theory (see Jusup *et al.* 2017b; Kooijman, 2010; Sousa *et al.*, 2008, 2010) is a formal metabolic theory offering a consistent mass- and energy-balanced framework for individuals and lower or higher levels of organization (Kooijman, 2010; Nisbet *et al.*, 2000). DEB models, based on the DEB theory, focus on energy acquisition and utilization throughout ontogeny as a function of environmental conditions. Depending on the organism and its ontogeny, there are three main groups of typified DEB models (Marques *et al.*, 2018). A standard DEB model, applied also here to the crayfish, is the simplest typified DEB model. It tracks the individual via three main state variables—reserve ($E$), structure ($V$) and maturity ($E_H$)—and the fourth state variable, reproduction buffer ($E_R$), which becomes relevant after puberty. Schematic representation and description of the model are given in Fig. 1, and specification and dynamics of state variables and energy fluxes are listed in Table 1, with parameters given in Table 2.

**Table 1:** State variables and dynamics of an individual and functions for temperature correction of metabolic rates. The 1-parameter correction ($c_T^{(1)}$) is used for correcting metabolic rates to temperatures within the temperature optimal niche, and the 5-parameter correction ($c_T^{(5)}$) is used for stress-inducing temperatures (for parameters, see Table 2).

**State variable (Units)**	**Description**	**Dynamics**
$ E$ (J)	Reserve energy	$\frac {d}{dt}E\,\,\,$ $=\dot {p}_A-\dot {p}_C$
$ V$ (cm$^3$)	Structural body volume	$\frac {d}{dt}V\,\,\,$ $=\frac {\dot {p}_G}{[E_G]}$
$ E_H$ (J)	Energy invested into maturation	$\frac {d}{dt}E_H$ $=\dot {p}_R(E_H<E_H^p)$
$ E_R$ (J)	Energy invested into reproduction	$\frac {d}{dt}E_R$ $= \dot {p}_R (E_H = E_H^p)$
**Process**	**Energy flux** (J.d$^{-1}$)	
Assimilation:	$ \dot {p}_A= \{\dot {p}_{Am} \}f L^2(E_H \ge E_H^b) $	
Mobilization:	$ \dot {p}_C = E \frac {\dot {v} [E_G] L^2+\dot {p}_S}{[E_G] L^3+\kappa E} $	
Somatic maintenance:	$ \dot {p}_S= {[\dot {p}_M]}L^3$	
Maturity maintenance:	$ \dot {p}_J = \dot {k}_J E_H$	
Growth:	$ \dot {p}_G = \kappa \dot {p}_C-\dot {p}_S$	
Maturation/reproduction:	$ \dot {p}_R = (1-\kappa )\dot {p}_C-\dot {p}_J $	
**Temperature correction** ^a^	***Equation***	***Comment***
1-parameter correction	$ c_T^{(1)} = \mbox {exp} \left (\frac {T_A}{T_{ref}} - \frac {T_A}{T} \right )$	Optimal temperature niche
5-parameter correction	$ c_T^{(5)} = c_T^{(1)} \left ( s_L^{ratio} + s_H^{ratio}\right ) \mbox {, with}$	Complete temperature niche
		(optimal and critical temps)
	$\> s_L^{ratio} = \frac { 1+ \mbox {exp}\left (\frac {T_{AL}}{T_{ref}} - \frac {T_{AL}}{T_L}\right ) } { 1+ \mbox {exp}\left (\frac {T_{AL}}{T} - \frac {T_{AL}}{T_L}\right )} \mbox {, and} $	applied for $(T \leq T_{ref})$
	$\; s_H^{ratio} = \frac { 1+ \mbox {exp}\left (\frac {T_{AH}}{T_H} - \frac {T_{AH}}{T_{ref}}\right )} {1+ \mbox {exp}\left (\frac {T_{AH}}{T_H} - \frac {T_{AH}}{T}\right )}.$	applied for $(T> T_{ref})$

^a^ coded as function tempcorr.m in software package DEBtool_M (DEBtool, 2021)

**Table 2:** List of primary and auxiliary parameters for the two endangered and two invasive species of crayfish: stone crayfish (*A. torrentium*), noble crayfish (*A. astacus*), signal crayfish (*P. leniusculus*) and marbled crayfish (*P. virginalis*). Parameters were estimated using the covariation method (Lika *et al.*, 2011; Marques *et al.*, 2019), unless noted otherwise. All rates are given at reference temperature $T_{ref}=293.15$ K (20$^{\circ }$C). The critical high and critical low temperatures are also expressed in $^{\circ }$C for easier readability.

Parameter description	Symbol	Unit	*A. torrentium*	*A. astacus*	*P. leniusculus*	*P. virginalis*
Max. assimilation rate^a^	$\left \{\dot {p}_{Am}\right \}$	J/d.cm$^{2}$	69.3065	192.025	253.508	158.437
Max. assimilation rate -males^a^	$\left \{\dot {p}_{Am}\right \}_m$	J/d.cm$^{2}$	84.1479	232.52	326.242	N/A
Energy conductance	$\dot {v}$	cm/d	0.04252	0.02944	0.03783	0.05713
Allocation fraction to soma	$\kappa $	-	0.9606	0.9305	0.9319	0.7051
Somatic maintenance rate	$[\dot {p}_M]$	J/d.cm$^{3}$	22.56	60.42	74.92	47.8
Specific cost for structure	$[E_G]$	J/cm$^{3}$	4443	4422	4374	4490
Maturity at hatching	$E_H^b$	J	1.261	1.053	1.963	0.077
Maturity at birth	$E_H^b$	J	2.031	2.435	2.042	6.709
Maturity at puberty	$E_H^p$	J	757.2	5844	8042	1735
Maturity at puberty - males	$E_H^p$	J	3614	3982	2976	N/A
Weibull aging acceleration	$\dot {h}_a$	1/d$^{2}$	1.018e-08	1.679e-08	8.963e-09	1.119e-07
Time at start development	$t_0$	d	63.56	43.28	30.94	22.89
Shape coeff.- total length	$\delta _M$	-	0.3132	0.2449	0.2513	0.2451
Shape coeff.- carapace length	$\delta _{MC}$	-	0.5553	0.5451	0.4948	0.5493
Shape coeff. at hatching	$\delta _{Mh}$	-	N/A	0.1607	N/A	0.1
Arrhenius temperature^b^	$T_A$	K	8000	8000	8000	8000
$T_A$ for high end of range^c^	$T_{AH}$	K	21000	21000	21000	21000
$T_A$ for low end of range^c^	$T_{AL}$	K	18000	18000	18000	18000
Temp. for high end of range^d^	$T_H$	K ($^{\circ }$C)	294.15 (21)	295.15 (22)	301.15 (28)	305.15 (32)
Temp. for low end of range^d^	$T_L$	K ($^{\circ }$C)	280.15 (7)	280.15 (7)	280.15 (7)	288.15 (15)
Typical temperature^d^	$T_{typ}$	K ($^{\circ }$C)	284.15 (11)	285.15 (12)	285.15 (12)	293.15 (20)

^a^ Indirectly estimated primary parameter, $\left \{\dot {p}_{Am}\right \}=L_{m}^{ref} z [\dot {p}_M]/ \kappa $ using the estimated values of $[\dot {p}_M]$, $\kappa $, and $z$ (or $z_m$ for males).^b^ Default value used for all species (AmP, 2021, Kooijman, 2010).^c^ Value adjusted manually to match the observed temperature response for *P.virginalis* (Fig. A.7); no data available for other species.^d^ Value set manually to match the literature (Jaklič *et al.*, 2014; Seitz *et al.*, 2005; Vogt, 2020). Other primary and auxiliary parameters (default values used): maximum searching rate, $\left \{\dot {F}_m\right \}=6.5\,$l d$^{-1}$ cm$^{-2}$; digestion efficiency (of food to reserve), $\kappa _X= 0.8$; defaecation efficiency (of food to faeces), $\kappa _P=0.1$; reproduction efficiency, $\kappa _R=0.95$; maturity maintenance rate coefficient, $\dot {k}_J=0.002$ d$^{-1}$; Gompertz stress coefficient, $s_G=0.0001$; density of structure and reserve, $d_V=d_E= 0.17$ g cm$^{-3}$.

**Table 3:** Life history data and corresponding model predictions for the two endangered and two invasive species of crayfish: stone crayfish (*A. torrentium*), noble crayfish (*A. astacus*), signal crayfish (*P. leniusculus*) and marble crayfish (*P. virginalis*). Reference for a specific data is listed - next to the corresponding enumerated superscript, in the table footnote, as well as any data-specific condition crucial for interpreting the data. ’Age at hatching’ corresponds to incubation time, and ’age at birth’ to incubation time + time until onset of feeding. All ages, including the life span, are expressed at a (species-specific) typical temperature (see Table 2), unless otherwise specified. Additional information relevant for model calibration is available in the Appendix. Predictions differing more than 10% (relative error) from data used as input are captured in boldface.

***Species*:**			***A. torrentium***		***A. astacus***		***P. leniusculus***		***P. virginalis***	
**Data:**		**Unit**	**Observed**	**Predicted**	**Observed**	**Predicted**	**Observed**	**Predicted**	**Observed**	**Predicted**
Age	Hatching	day	210^(1)^	207.3	154^(1)^	147.5	70^(1)^	72.09	20^(1)^	**24.71**
	Birth	day	220^(1)^	223	161^(2)^	164.4	80^(2)^	76.17	45^(1)^	**33.46**
	Puberty ($\female $)	day	1095^(2)^	1119	1460^(3,4)^	**1186**	1095^(3)^	1116	119^(2)^	132.9
	Puberty ($\male $)	day	-	-	1095^(3)^	1095	730^(3)^	729.8	N/A	N/A
Life span		day	7300^(3)^	7300	6570^(4,5)^	6333	7300^(4)^	7335	1160^(3)^	1160
Carapace length	Hatching	cm	0.34^(4)^	0.3414	-	-	-	-	-	-
	Puberty ($\female $)	cm	2.6^(4)^	2.59	-	-	-	-	-	-
	Ultimate ($\female $)	cm	-	-	-	-	-	-	4.5	4.255
Total length	Hatching	cm	-	-	0.9^(4)^	0.9009	-	-	0.35^(1)^	0.3448
	Birth	cm	-	-	1.2^(4)^	1.181	1.1^(2)^	**0.7212**	0.57^(1)^	0.6172
	Puberty ($\female $)	cm	6^(5)^	**4.792**	7.7^(3,4)^	7.71	8^(4)^	8.202	3.5^(1,2)^	3.529
	Puberty ($\male $)	cm	-	-	-	-	8.2^(3)^	8.24	N/A	N/A
	2-yr old ($\female $)	cm	-	-	4.7^(3)^	**5.453**	7.9^(3)^	**6.405**		
	2-yr old ($\male $)	cm	-	-	4.9^(3)^	**6.022**	8.2^(3)^	7.458	N/A	N/A
	Ultimate ($\female $)	cm	9.7^(5,6)^	9.422	12^(4)^	12.07	12^(5)^	12.55	10^(2,4)^	9.536
	Ultimate ($\male $)	cm	10.5^(5,6)^	11.44	16^(4)^	14.62	16^(5)^	16.15	N/A	N/A
Wet weight	Hatching	g	-	-	-	-	0.01575^(2)^	0.01554	-	-
	Birth	g	-	-	0.014^(3)^	**0.01826**	0.035^(2)^	**0.01615**	0.0053^(5)^	**0.005915**
	Puberty ($\female $)	g	-	-	18^(3,6)^	17.96	29^(3,6)^	**23.75**	1.537^(5)^	**1.106**
	Ultimate ($\female $)	g	40^(5)^	36.4	68.2^(6)^	69	89.4^(3,6)^	85.08	23^(2,3,4)^	21.82
	Ultimate ($\male $)	g	70^(5)^	69.28	161.5^(6)^	**138.7**	212^(3,6)^	214.2	N/A	N/A
Maximum reproduction rate^(a)^		Eggs/day	0.1096^(1,2,7)^	**0.127**	0.5479^(4,7)^	0.5792	0.9589^(4,7)^	0.9796	5.4795^(1,4,6)^	**7.356**
Initial energy content of egg^(b)^		Joule	70.08^(8)^	70.13	-	-	64.07^(8)^	70.16	37.54^(1)^	**32.52**

^(a)^Reproduction assumed to occur at species-specific frequencies, abundant food and typical (species-specific) temperature listed for each species.^(b)^Energy content of redclaw crayfish (Rodríguez-González *et al.*, 2006) used as reference value, and scaled to egg size of corresponding species, assuming same energy content per egg volume. Data sources and most relevant notes for each crayfish species are as follows: *A. torrentium*: ^(1)^Maguire *et al.* (2002) - data from the wild; ^(2)^Parvulescu (2019); ^(3)^Holdich (2002) - data for other freshwater crayfish; ^(4)^Huber & Schubart (2005); ^(5)^Dakic & Maguire (2016), Maguire *et al.* (2002); ^(6)^Vlach & Valdmanova (2015); ^(7)^ Encyclopedia of Life. Calculated as 80 eggs and reproduction frequency every second year at 11$^{\circ }$C and abundant food, more details in Appendix; ^(8)^Maguire *et al.* (2005)*A. astacus*: ^(1)^Policar *et al.* (2004) - data from laboratory, average temp 10.45$^{\circ }$C, without cold diapause; ^(2)^Hessen *et al.* (1987); ^(3)^Abrahamsson (1971); ^(4)^Holdich (2002); ^(5)^Souty-Grosset *et al.* (2006); ^(6)^Maguire *et al.* (2004); ^(7)^ Calculated as 200 eggs every year, at 11$^{\circ }$C.*P. leniusculus*: ^(1)^Celada *et al.* (1987) - data from laboratory, incubation temp 15.5$^{\circ }$C, without cold diapause; ^(2)^Kozák *et al.* (2009); ^(3)^Abrahamsson (1971); ^(4)^Souty-Grosset *et al.* (2006); ^(5)^Koese & M., S. (2011); ^(6)^Westman & Savolainen (2002); ^(7)^ Calculated as 350 eggs every year, at 11$^{\circ }$C. ^(8)^Pawlos *et al.* (2010) *P. virginalis*: ^(1)^Vogt *et al.* (2004); ^(2)^Kouba *et al.* (2021); ^(3)^Vogt (2010); ^(4)^Vogt *et al.* (2015); ^(5)^Seitz *et al.* (2005); ^(6)^Hossain *et al.* (2019), Vogt *et al.* (2019). Calculated as 400 eggs every 70 days (5 reproduction cycles per year), at 20$^{\circ }$C.

The standard DEB model assumes two major metabolic transition points: ‘birth’, defined as the transition from embryo (does not feed and does not invest energy into reproduction) to juvenile (feeds but does not invest energy into reproduction); and ‘puberty’, defined as the transition from juvenile to adult (feeds and invests energy into reproduction). Progression through these DEB-defined life stages is tracked via the maturity state variable ($E_H$), where metabolic switches occur when energy invested into maturity reaches a corresponding threshold: $E_H^b$ for birth and $E_H^p$ for puberty.

Life cycle of some (marine) decapod crustaceans includes a larval phase and a metamorphosis to juvenile form (Holdich, 2002). The larval phase and the subsequent metamorphosis often coincide with a metabolic acceleration, i.e., a gradual increase in selected parameter values occurring between birth and metamorphosis (Kooijman *et al.*, 2011). Such a transition typically calls for an extension of the standard DEB model (Marques *et al.*, 2018). Freshwater crayfish, however, are somewhat specific and lack this phase: postembryonic development is terminated by the development of the independent (freely moving and exogenously feeding) juveniles, which are morphologically similar to their parents (Holdich, 2002). This motivated us to use the standard DEB model, with the intention to expand it if data indicate the necessity.

Temperature affects metabolic rates. DEB models account for the effects by multiplying all metabolic rates by the Arrhenius temperature correction function (Kooijman, 2010, Section 1.3; Schoolfield *et al.*, 1981), marked here as $c_T$. We consider two forms of the function (Table 1): 1-parameter correction $c_T^{(1)}$ for correcting metabolic rates within the species optimum temperature niche and 5-parameter correction $c_T^{(5)}$ when rates are corrected to a temperature-inducing organismal stress. Values for the optimal temperature niche were taken from the literature: 11–17$^{\circ }$C for *A. torrentium*, 12–20$^{\circ }$C for *A. astacus*, 10–24$^{\circ }$C for *P. leniusculus* and 18–26$^{\circ }$C for *P. virginalis* (Jaklič *et al.*, 2014; Vogt, 2020). Stress-inducing temperatures were assumed to be several degrees outside of the optimal temperature niche and, based on available information, were defined as follows: 7$^{\circ }$C for Astacidae and 15$^{\circ }$C for *P. virginalis* as low temperatures and 21$^{\circ }$C for *A. torrentium*, 22$^{\circ }$C for *A. astacus*, 28$^{\circ }$C for *P. leniusculus* and 32$^{\circ }$C for *P. virginalis* as high temperatures (Table 2).

Food availability is modeled as a scaled functional response ranging from zero (no food) to 1 (abundant food), i.e. it is a saturating function of food abundance

(1) \begin{align*}& f = \frac{X}{X+K_X}, \end{align*}

where $X$ is an abundance of food of certain quality and $K_X$ is the half-saturation constant defined by the metabolism of the individual: $ K_X = \left \{\dot {p}_{Am} \right \}/ (\kappa _X \cdot \dot {F}_m)$ (for parameter description and values, see Table 2; for additional notes on $f,$ the reader is referred to Appendix A).

**Fig. A.1:**
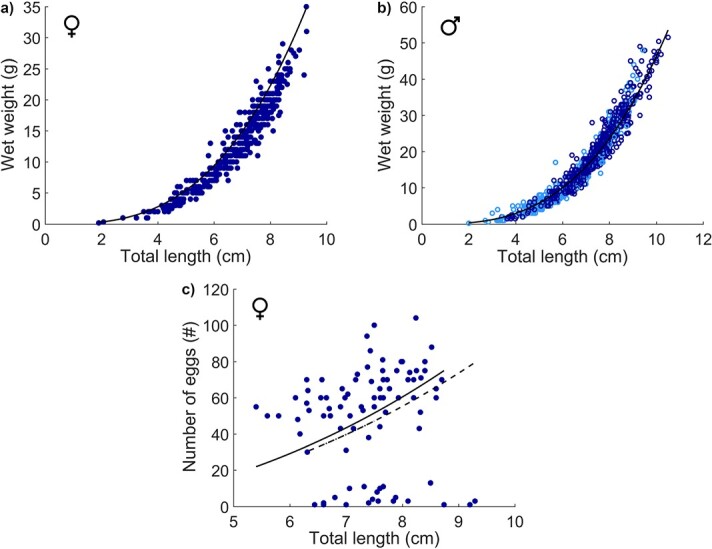
Stone crayfish, *Austropotamobius torrentium* - data (markers) and model predictions (lines). Length-weight relationship, and fecundity as a function of length. Data sources: (a) and (c) Maguire & Klobučar (2011); (b) Maguire & Klobučar (2011) (dark blue symbols) and Berger *et al.* (2018) (light blue symbols). Relevant parameters for model predictions are given in Table 2. Scaled functional response for all datasets is 1.

Basic equations (Table 1) are the same for all four crayfish species; models only differ in the parameter values. We estimated parameter values using published data on life history traits (Table 3) and datasets on growth, reproduction and length–weight relationships (Table 4; see also Figs A.1–A.7 in the Appendix). Special focus was given to datasets containing information on metabolic performance (e.g. growth) at different temperatures or at food of different quality and/or quantity. Parameters were estimated separately for each species using MATLAB, by running the freely available software package AmPtool (AmPtool, 2021) and routines in its supporting package DEBtool_M (DEBtool, 2021). Parameter descriptions and values are listed in Table 2. Parameterized models were assessed for their goodness of fit and subsequently used in further simulations (see next section).

**Table 4:** Uni-variate data for the two endangered and two invasive species of crayfish: stone crayfish (*A. torrentium*), noble crayfish (*A. astacus*), signal crayfish (*P. leniusculus*) and marble crayfish (*P. virginalis*). Symbols: T - temperature; $f$ - food; t - time; TL - total length; CL - carapace length; Ww - wet weight; N - fecundity.

**Data type:**	***A. torrentium***	***A. astacus***	***P. leniusculus***	***P. virginalis***
Time - Length				t – TL
at different T			t – TL_T	t – CL_T
at different $f$		t – TL_$f$	t – CL_$f$	
Time - Wet weight				
at different T			t – Ww_T	t – Ww_T
at different $f$		t – Ww_$f$	t – Ww_$f$	
Length - Wet weight	TL – Ww	TL – Ww	TL – Ww	TL – Ww
		CL – Ww		
Length - Fecundity		TL – N		TL – N
at different T	TL – N_T		TL – N_T	
Wet weight - Fecundity				Ww – N

Data sources for each crayfish species: *A. torrentium*: Berger *et al.* (2018); Maguire *et al.* (2002); Maguire & Klobučar, (2011); Maguire *et al.* (2005); *A. astacus*: (Abrahamsson (1971); Basta (2014); Hudina *et al.* (2011b); Maguire *et al.* (2004); Policar *et al.* (2004); Westman & Pursiainen (1982)); *P. leniusculus*: (Abrahamsson (1971); Belchier *et al.* (1998); González *et al.* (2010); Hudina *et al.* (2011c); Kozák *et al.* (2009); Martinsson (2011); Westman & Savolainen (2002); *P. virginalis*: (Hossain *et al.* (2019); Parvulescu *et al.* (2017); Seitz *et al.* (2005); Velisek *et al.* (2014); Vogt (2010); Vogt *et al.* (2019, 2008); žižak (2015).

### 2.3 Simulations of food availability and temperature

Model simulations were performed with the main goal to analyse responses of the four crayfish species to different levels of food availability and different temperature. To maximally simplify the results interpretation and enable direct comparison of the model predictions, we focused on an average individual of each species, as represented by the DEB model, and on two aspects of the life cycle: size and reproduction. Size was tracked as total length and reproduction as reproductive output either in one reproductive event, or cumulative annual reproductive output. Simultaneously, we marked the size and age of the individuals when they reached puberty in the simulations.

Food availability was modeled via the scaled functional response, $f$. As mentioned earlier (Eq. 1), $f$ expresses food density scaled by the half-saturation coefficient, which is species specific. For each species, we simulated a constant $f$ ranging from the minimum $f$ enabling the individual to sexually mature ($fp_{min}$; species-specific value) to $f=1.1$. By definition, values of $f$ can range between 0 (no food) and 1 (*ad libitum* food), when food quality does not change; in extreme cases when food quality drastically increases relative to the reference food source, $f\!>\!1$ is possible. Temperature was for these simulations unchanged and was a constant 12.5$^{\circ }$C (IKSR, 2013; Maguire & Gottstein-Matočec, 2004; see below).

Temperature simulations were based on the RCP scenarios for temperature increase (IPCC, 2013; Knutti & Sedláček, 2013), relative to the baseline temperature. We chose 12.5$^{\circ }$C as baseline temperature for all crayfish species, as this is close to the average water temperature of (i) the largest river basin in continental Europe (Rhine river basin, 12.6$^{\circ }$C; IKSR, 2013) and (ii) various water bodies throughout different biogeographical regions of Croatia (Continental, Alpine, Pannonian and Mediterranean, 12.3$^{\circ }$C; Maguire & Gottstein-Matočec, 2004). All four studied species occur either as native or invasive species in these areas (Kouba *et al.*, 2014). Simulated increase of temperature ranged from 1.5 to 4.5 degrees, to include projections by all RCP scenarios: from those expecting a low increase in greenhouse gas emissions (RCP 2.6, mean surface temperature increase of 1.5 degree) to those accounting for a high increase in greenhouse gas emissions (RCP 8.5, mean surface temperature increase of 4.5 degrees) (IPCC, 2013; Knutti & Sedláček, 2013). The temperature increase could, on one hand, cause thermal stress for native species preferring colder waters, and on the other hand, improve conditions for invasive species preferring warmer waters (Jaklič *et al.*, 2014; Vogt, 2020). Therefore, the $c_T^{(5)}$ temperature correction was used for the simulations. Food availability for these simulations was for all species approximated by the reference scaled functional response $f=1$.

For species sharing the same ecological niche—the endangered native *A. astacus* and the invasive *P. leniusculus*—we additionally simulated a relative change in actual food availability. Food density ($X$) was first expressed relative to the native crayfish (*A. astacus*), and then used to calculate the scaled functional response of the invader (*P. leniusculus*) using (Eq. 1) and parameter values estimated for *P. leniusculus*. Then we decreased the food density by 20% and recalculated respective $f$ values. For these simulations, we used $f=0.8$ as proxy for the initial food availability, because (i) food densities resulting in values much higher than 0.8 need to be extremely large due to the asymptotic shape of the $f$ function, and therefore a decrease in $X$ would need to be unrealistically drastic ($>\!\!\!80\%$), and (ii) food availability of individuals in the wild has been estimated to be $f=0.8$ (e.g., Marn *et al.* 2017b). Temperature has either been simulated as 12.5$^{\circ }$C to match the applied baseline temperature (which is also within the optimal thermal range for both species; Jaklič *et al.*, 2014), or as a 2 degree temperature increase. In total, four scenarios were simulated: (i) *control*: food availability resulting in $f=0.8$ for *A. astacus* and environmental temperature of 12.5$^{\circ }$C; (ii) *food*: food density decreased by 20% (temperature unchanged); (iii) *temperature*: temperature increased by 2 degrees (food density unchanged); and (iv) *combined*: food density decreased by 20% and temperature increased by 2 degrees.

## 3 Results

**Fig. 2:**
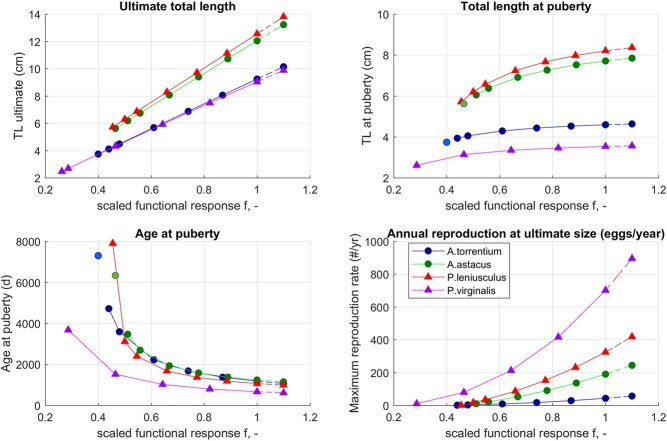
Simulation for different food availability modeled as scaled functional response, $fp_{min} \leq f \leq 1.1$, at baseline temperature of 12.5$^{\circ }$C). Different colors and markers denote different species: native species are plotted with circles and invasive species with triangles. Reproduction of all crayfish is expressed on annual basis to enable direct comparison: we use the reproduction of *A. torrentium* (simulated as once every 2 years; Parvulescu, 2019) and reproduction of *P. virginalis* (simulated as five times per year; Vogt *et al.*, 2004) to calculate the reproductive output per year. The results interpretation focuses on simulations with $f \leq 1$; higher $f$ settings are indicated by dashed lines in the figures. For *A. torrentium* and *A. astacus* puberty was assumed to occur just prior to death at the lowest simulated food setting (indicated by a disconnected, lighter shaded marker in the top right and the bottom left panel).

**Fig. 3:**
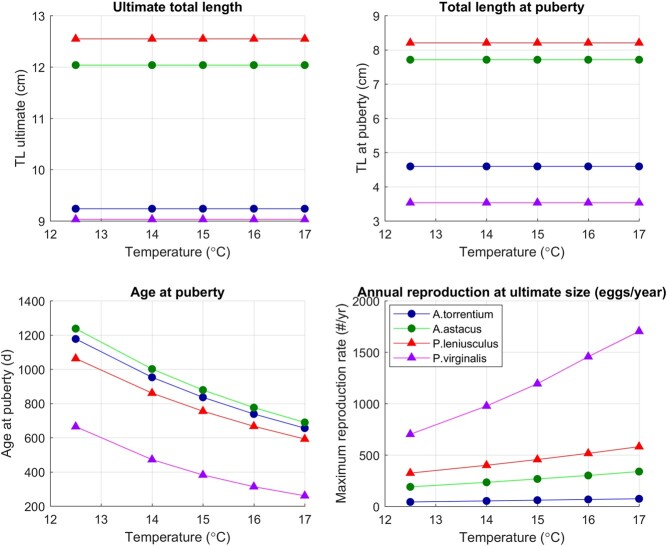
Simulation for current conditions (baseline temperature of 12.5$^{\circ }$C) and a temperature increase of 1.5–4.5 degrees. Different colors and markers denote different species: native species are plotted with circles and invasive species with triangles. Reproduction of all crayfish is expressed on annual basis to enable direct comparison: we used the predicted reproduction of *A. torrentium* (simulated as once every 2 years; Parvulescu, 2019) and reproduction of *P. virginalis* (simulated as five times per year; Vogt *et al.*, 2004) to calculate the reproductive output per year.

**Fig. 4:**
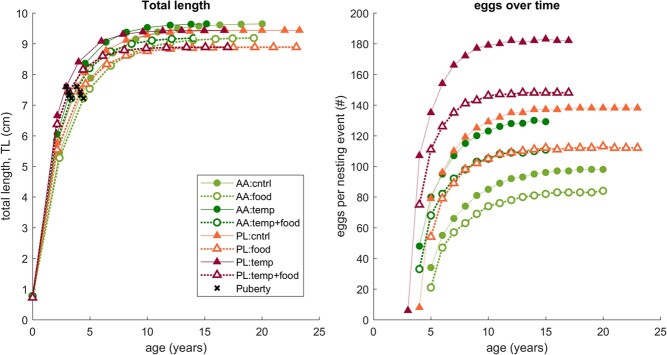
Comparing the performance of two physiologically most similar species—the native *Astacus astacus* (AA, green circles) and the invasive *Pacifastacus leniusculus* (PL, red triangles)—under four scenarios. Simulated scenarios are as follows: (i) *control*: food availability resulting in $f=0.8$ for AA and $f=0.75$ for PL and environmental temperature of 12.5$^{\circ }$C; (ii) *food*: food density decreased by 20%, resulting in $f=0.76$ for AA and $f=0.71$ for PL (temperature unchanged); (iii) *temp*: temperature increased by 2 degrees (food density unchanged); and (iv) *combined*: food density decreased by 20% and temperature increased by 2 degrees. Food decrease is denoted by empty symbols, and temperature increase by darker shade of color.

Generally, the models describe well the life cycle and life history traits of all four studied species (Table 3). The model parameters are in broad agreement with DEB parameters of other decapod species (AmP, 2021) and, when studied in more detail, point to phylogenetic differences among the four studied crayfish (Table 2). The model predictions for uni-variate data matched the data used for model parameterization for all four species (Table 4; Figs A.1–A.7 in the Appendix). Simulations of different scaled food availability ($f$) identified that smaller species (*A. torrentium* and *P. virginalis*) can reach puberty at a lower value of $f$ than larger species (*A. astacus* and *P. leniusculus*). Within the relevant $f$ range, Astacidae have a more moderate response than *P. virginalis* (Fig. 2). Simulations of a temperature increase produced a similar response among cold-water species (*A. torrentium*, *A. astacus* and *P. leniusculus*), and a more pronounced response of the warm-water species (*P. virginalis*), which in the scenario with a larger temperature increase reached its thermal optimum (Fig. 3). Directly comparing the performance of the two ecologically most similar crayfish — the native noble crayfish, *A. astacus*, and the invasive signal crayfish, *P. leniusculus* — in the same environment (characterized by temperature and the absolute amount of some food), we identified a metabolism-driven difference in the perceived food availability, resulting in slower growth of the invader, but not offsetting its higher reproductive output (Fig. 4). We discuss the results in more detail below.

### 3.1 Model parameters

Estimation of model parameters was performed separately for each species and produced four sets of parameter values (Table 2). Generally, all four crayfish have similar values of specific cost for structure ($[E_G]$). The phylogenetically more related Astacidae (*A. torrentium*, *A. astacus* and *P. leniusculus*) all share a relatively high value of $\kappa $, indicating a preferential energy investment into maintenance and growth over maturation and reproduction. The highest value of $\kappa = 0.96$ is estimated for the native *A. torrentium,* which could explain its low reproductive output, despite the low maturity level at puberty ($E_H^p$) and thus low maturity maintenance post-puberty (see Table 1 for energy fluxes). The invasive *P. virginalis*, by contrast, has a relatively low $\kappa $ and thus invests roughly 30% of all available energy ($1-\kappa =0.295$) into its maturation and reproduction. This results in a much earlier age at birth and puberty, and a larger reproductive output compared with the other three species. Maturity levels for birth ($E_H^b$) and puberty ($E_H^p$) are generally similar among Astacidae. The exception is a relatively low $E_H^p$ value estimated for the (female) *A. torrentium*, possibly coupled with a low value for males (currently not well defined by data as information on age and/or length at puberty was not available for males; Table 3).

Among the four studied crayfish, *A. torrentium* has the smallest assimilation potential ($\{\dot {p}_{Am}\}$; see Table 2), which is reflected in its smaller ultimate size (Table 3). *P. virginalis* also reaches a smaller ultimate size; however, the possible explanation is a relatively small $\kappa $ limiting its ultimate size, rather than its assimilation potential, which is roughly double that of *A. torrentium* and comparable with that of a larger noble crayfish.

Parameters used for the temperature correction (last section of Table 2) are the same among all four species, with the exception of critical low and high temperatures, which are species specific and were taken from literature. In principle, the default value of the Arrhenius temperature ($T_A$) and the 1-parameter correction factor $c_T^{(1)}$ were sufficient to correct the metabolic rates and obtain satisfactory predictions for all data obtained within the optimum temperature niche (see Table 1 for expressions and Table 3 and Figs A.1–A.7 for fits of model predictions to data). The exception is *P. virginalis*, for which growth was recorded also at temperatures causing stress (Fig. A.7). This required a more complex 5-parameter temperature correction function and enabled the parameterization of all five parameters needed for computing $c_T^{(5)}$.

### 3.2 Fits of model predictions to observed data

The overall goodness of fit is numerically evaluated in the AmPtool routines by calculating MRE (Mean Relative Error) and SMSE (Standard Mean Squared Error); both measures have a theoretical range between zero (perfect fit) and 1 (Marques *et al.*, 2019). For studied species, the goodness of fit values are as follows: MRE = 0.137 and SMSE = 0.183 for *A. torrentium*; MRE = 0.103 and SMSE = 0.111 for *A. astacus*; MRE = 0.132 and SMSE = 0.160 for *P. leniusculus*; and MRE = 0.186; SMSE = 0.212 for *P. virginalis*. Generally, goodness of fit deteriorates when more data types are included (AmP, 2021: About section), but more data types are encouraged to better define parameter values. Variety of data types is marked by the completeness level (1–10; Lika *et al.*, 2011). For our species the completeness ranges from 2.5 (*A. torrentium*) to 3.2 (*A. astacus*) and 3.5 (*P. leniusculus* and *P. virginalis*), which is above the average for the whole Add-my-Pet collection (2.3; AmP, 2021: About section).

During parameter estimation, all data are simultaneously taken into account to determine parameter values (Marques *et al.*, 2019). We gave more emphasis to selected life history traits used in further simulations (ultimate length, length and age at puberty, reproductive output at ultimate size) and growth and reproduction datasets obtained at several levels of food or several temperatures. This, however, did not worsen the fits between other data and predictions, resulting overall in very good agreement between model predictions and data for all four species and all datasets. Data for 2-year-old males and females have >10% relative error (RE; Table 3) and should probably be excluded from the models. Data was extracted from histograms (Abrahamsson, 1971, Fig.1) and, while somewhat informative in the previous version of the *A. astacus* and *P. leniusculus* models (AmP, 2021, archived versions), it has now been replaced with more reliable data. Predictions for *A. astacus* male ultimate length and weight have a relatively high error compared with observations and are also not ideal for *A. torrentium*. For females, all traits were predicted very well (<10% RE) for all species.

Model predictions for various uni-variate datasets (Table 4) are presented in the Appendix due to high number of figures in the manuscript. (Additionally, results and files, including detailed referenced data sources and corresponding temperatures used to obtain model predictions, can be viewed and downloaded online in the Add-my-Pet DEB database; see AmP, 2021.) For **stone crayfish, *A. torrentium***, model predictions match length–weight curves of males and females very well, with RE ranging between 9% and 18%. Predictions for the relationship between female length and number of eggs match the observed trend, but have a higher RE (40%–60%) due to females with uncharacteristically few eggs (Fig. A.1).

**Fig. A.2:**
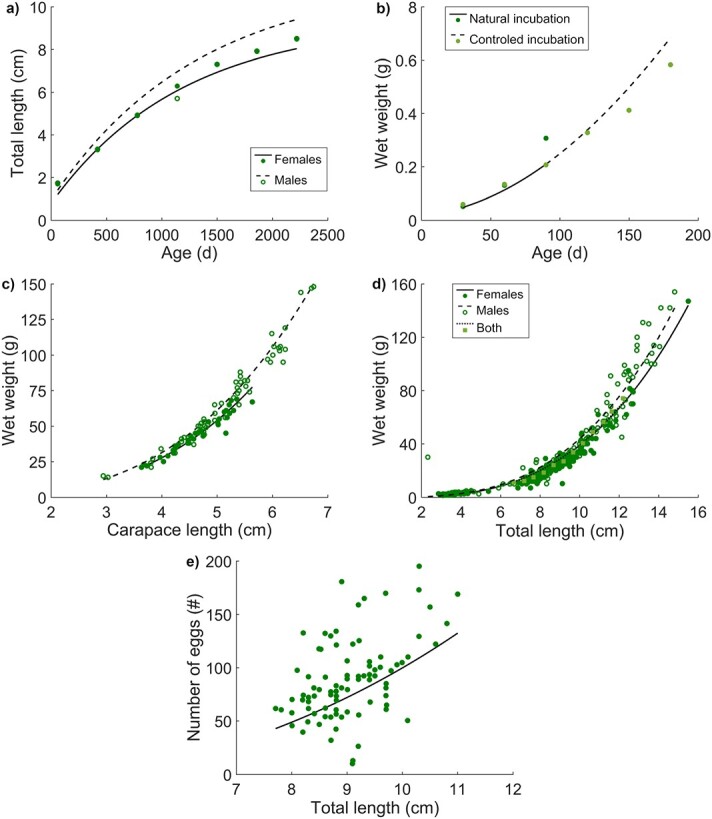
Noble crayfish, *Astacus astacus* - data (markers) and model predictions (lines). Growth in length and weight, length-weight relationship, and fecundity as a function of length. Data sources: (a) Westman & Pursiainen (1982); (b) Policar *et al.* (2004); (c) Hudina *et al.* (2011b); (d) Abrahamsson (1971) and Maguire *et al.* (2004), combined; (e) Abrahamsson (1971). Relevant parameters for model predictions are given in Table 2. Dataset specific scaled functional responses are as follows: Westman & Pursiainen (1982) - 0.7751; Policar *et al.* (2004) - 0.5504; Hudina *et al.* (2011b) and Abrahamsson (1971) - 1; Maguire *et al.* (2004) - 0.9777.

Length–weight data for male and female **noble crayfish, *A. astacus*** are also predicted well by the model, with a small RE (6% to 17%), and length–fecundity trend is predicted well, but the RE is relatively large (30%). Age–length and age–weight relationships are predicted well for females and males, even when accounting for food of different quality experienced under two population densities: age–length predictions with RE $\leq 10$% and age–weight with RE 9% to 24% (Figs A.2 and A.3).

**Fig. A.4:**
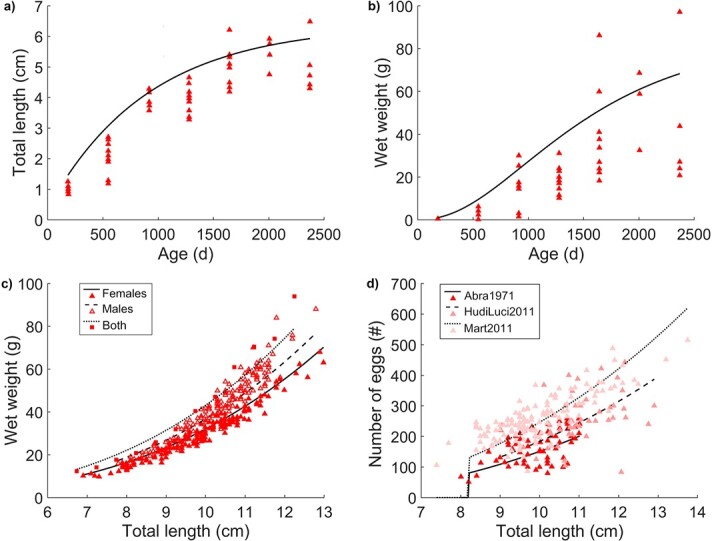
Signal crayfish, *Pacifastacus leniusculus* - data (markers) and model predictions (lines). Growth in length and weight, length-weight relationship, and fecundity as a function of length. Data sources: (a) and (b) Belchier *et al.* (1998); (c) Abrahamsson (1971) (squares; dotted line) and Westman & Savolainen (2002) (triangles; solid and dashed lines); (d) Abrahamsson (1971), Hudina *et al.* (2011c) and Martinsson (2011). Relevant parameters for model predictions are given in Table 2. Dataset specific scaled functional responses are as follows: Westman & Savolainen (2002) - 0.5950; All other datasets - 1.

Model predictions for **signal crayfish, *P. leniusculus*** matched the data in a similar way: length–weight data is predicted very well for males and females (RE $\leq $ 10%) and the length–fecundity trend is matched well but data variability incur a relatively high RE (17% to 25%). Age–length and age–wet weight relationships are also predicted well for nine data sets (RE 7% to 20%), but RE for two datasets is higher (20% and 30%), probably due to data scatter. Observed differences in growth—due to different food levels and rearing temperatures—are also predicted well (Figs A.4 and A.5).

**Fig. A.6:**
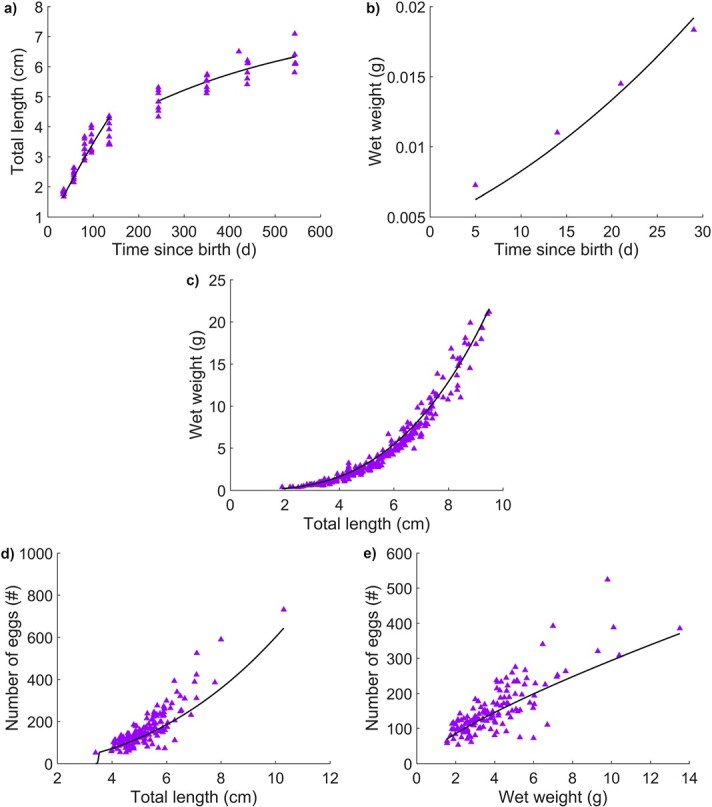
Marble crayfish, *Procambarus virginalis* - data (markers) and model predictions (lines). Growth in length and weight, length-weight relationship, and fecundity as function of length and of weight. Data sources: (a) Vogt *et al.* (2008); (b) Velisek *et al.* (2014); (c) Vogt (2010), žižak (2015), Parvulescu *et al.* (2017) and Hossain *et al.* (2019), combined; (d) Hossain *et al.* (2019) and Vogt *et al.* (2019), combined; (e) Hossain *et al.* (2019). Relevant parameters for model predictions are given in Table 2. Dataset specific scaled functional responses are as follows: Vogt *et al.* (2008) - 1.5 and 0.7709; Velisek *et al.* (2014) - 0.462; Scaled functional response for all other datasets is 1.

**Fig. A.7:**
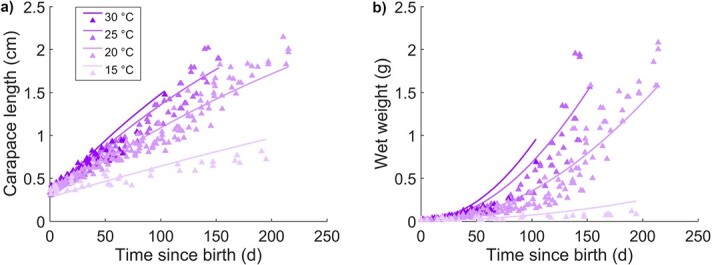
Marble crayfish, *Procambarus virginalis* - data (markers) and model predictions (lines). Carapace length and wet weight vs time since birth at different temperatures. Data source: Seitz *et al.* (2005). Relevant parameters for model predictions are given in Table 2. Dataset specific scaled functional response is 0.9161.

Finally, **marbled crayfish, *P. virginalis*** length–weight predictions match the data trend with RE ranging from 3% to 20%. Fecundity is predicted well with RE 26% for length–fecundity and RE 20% for weight–fecundity data. Growth in length is predicted very well within the temperature optimum niche (20$^{\circ }$C and 25$^{\circ }$C; RE $\leq $10%), and somewhat poorer (but still well) at lower and higher temperature outside the optimal niche (RE $\approx $16%). Growth in weight, although in principle predicted well by the model, has the highest RE of all datasets: $\approx $ 30% within the temperature optimum niche, 74% at 30$^{\circ }$C and 93% at 15$^{\circ }$C. However, even with relatively high RE, the model predictions match the slower-than-expected growth at the temperature extremes (Figs A.6 and A.7).

### 3.3 Results of food availability and temperature simulations

Simulations were performed for males and females, but we present results for females only. We decided this because (i) model predictions are more accurate for females than for males—traits of interest (ultimate length and length and age at puberty) for females of all species were predicted very well (RE < 10%, Table 3), whereas for males the data were either not available or are predicted poorer (with the exception of *P. leniusculus*)—and (ii) reproduction data were available only for females.

**Fig. B.8:**
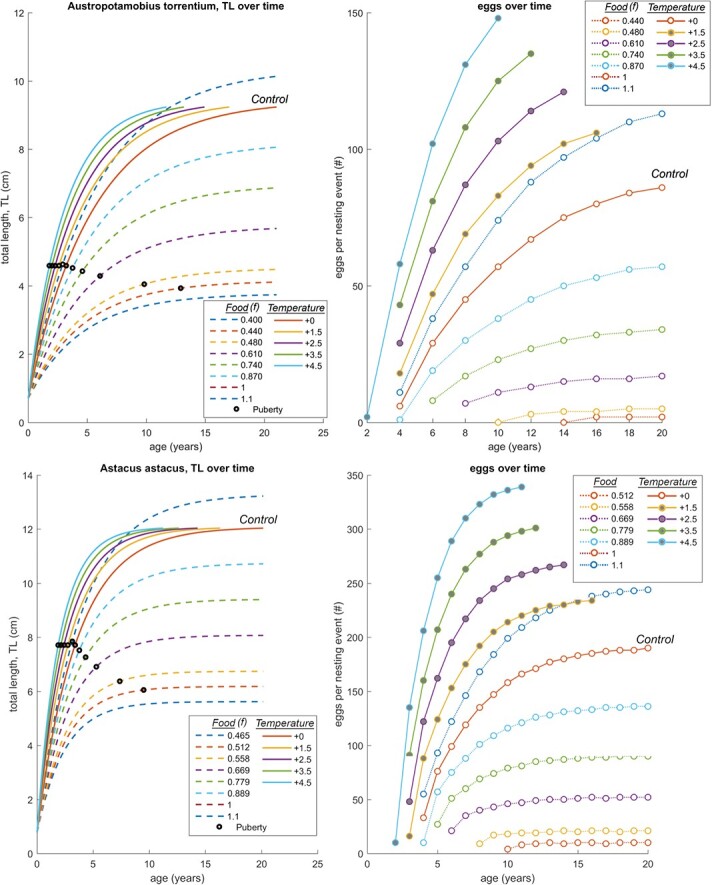
Simulations of different food quality and quantity, and different levels of temperature increase, for stone crayfish (*Austropotamobius torrentium*) and noble crayfish (*Astacus astacus*). Reproduction of stone crayfish is biennial (every two years, Parvulescu, 2019), and that of noble crayfish is annual. Food simulations are depicted with dashed lines and empty circles, and temperature simulations with solid lines and full circles. For all food simulations, the temperature is 12.C$^{\circ }$C; for all temperature simulations, the food is simulated as $f=1$; the combination of T = 12.5$^{\circ }$C and $f=1$ is marked as ’control’.

**Fig. B.10:**
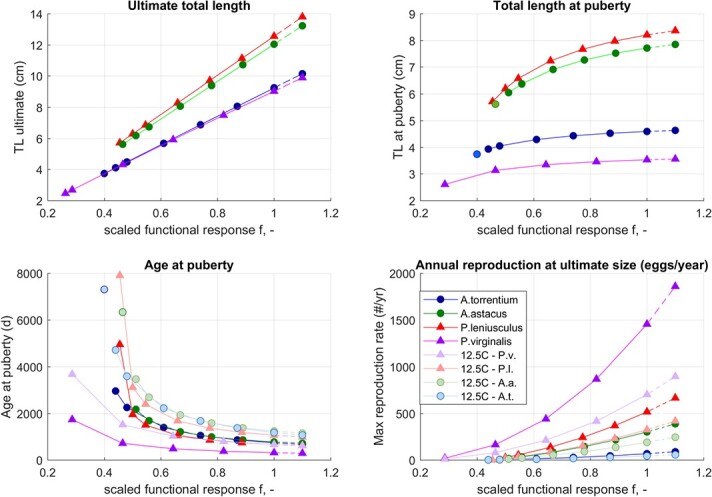
Simulation for different food availability, modeled as scaled functional response $f$. Different colors and markers denote different species: native species are plotted with circles and invasive species with triangles. Reproduction of all crayfish is expressed on annual basis to enable direct comparison: we used the reproduction of *A. torrentium* (simulated as once every two years, Parvulescu, 2019), and reproduction of *P. virginalis* (simulated as five times per year, Vogt *et al.*, 2004) to calculate the reproductive output per year. Light colors are the simulations at the baseline temperature of 12.5$^{\circ }$C (described in the main text), and more intense colors the additional simulations with 16$^{\circ }$C set as baseline temperature (in the top two panels the lines overlap). The results interpretation focuses on simulations with $f \leq 1$; higher $f$ setting is indicated by dashed lines. For *A. torrentium* and *A. astacus* puberty was assumed to occur just prior to death at the lowest simulated food setting (indicated by disconnected markers in the top right and the bottom left panel).

We present an overview of simulation results in the main text (Figs 2 and 3), accompanied by more detailed results for each species in the Appendix (Figs B.8 and B.9; see also Figs B.10 and B.11). Theoretical ultimate length, determined by the combination of species parameters, $L_m = \kappa \{\dot {p}_{Am}\} / [\dot {p}_M]$, and the scaled functional response for food ($f$-value), was in some cases larger than total length reached during an individual’s life time. In such cases, realized rather than theoretical values are presented in figures and used in analysis. Even though the figures include the complete range of simulated $f$ values (up to $f=1.1$), we analyse in more detail results obtained within the ecologically more relevant range ($fp_{min} < f< 1$), where $fp_{min}$ is the minimum $f$ value required to reach puberty. In some cases, $fp_{min}$—a species specific (theoretical) $f$-value obtained by DEBtool_routine statistics_st—was too low for some species to reach puberty, i.e. the model predicted age at puberty, which exceeds the maximum life span. In such cases, figures lack a prediction point for the corresponding $f$ value, but for simplicity, for the purpose of the analysis, we assumed that puberty is reached shortly prior to death at $fp_{min}$.

***Effects of food availability*** Simulations of length, maturation and reproduction at a range of $f$ values (simulated $fp_{min} \geq f \leq 1.1$) showed that all selected life history traits are affected by food availability. Effects varied between species (Fig. 2 in the main article and Figs B.8 and B.9 in the Appendix). Larger-sized species (*P. leniusculus* and *A. astacus*) are able to reach puberty and reproduce within a narrower range of $f$ values. Within biologically realistic range, $fp_{min} \leq f \leq 1$ the ultimate size increased by 114% for *A. astacus* and by 120% for *P. leniusculus*. Smaller *A. torrentium* increased in ultimate size by $\approx $ 150 %, while the super-invader *P. virginalis* can increase its ultimate length by astonishing $260$%.

Assuming that puberty is reached shortly prior to death at $fp_{min}$, length at puberty increased by $\approx $ 40% for *A. astacus* and *P. leniusculus* crayfish, and by 23% for *A. torrentium*. For *P. virginalis* length at puberty increased by 35% (comparing $L_p$ at $fp_{min}$ and $f=1$; Fig. 2). Age at puberty decreased from equaling the life span to 19.5% of the life span for *A. astacus*, 13.5% of the life span for *P. leniusculus*, $\approx $16% of the life span for *A. torrentium* and 18% of the life span for *P. virginalis*. At the simulated temperature of 12.5$^{\circ }$C and $f=1$ absolute values of age at puberty were for all four species within a much narrower range than at $fp_{min}$, and ranged between 664 days (*P. virginalis*) and 1237 days (*A. astacus*). Note that the temperature has a strong effect on all rates, so the predicted values do not necessarily correspond to those observed in nature where the temperature is not the constant 12.5$^{\circ }$C simulated here; see also Fig. 3.

Reproduction at ultimate size increased for all species as a function of $f$, but resulted in drastically different annual reproductive output between species: for example, at $f\approx 0.7$ (and 12.5$^{\circ }$C), *A. torrentium* produced only about 15 eggs per year, *A. astacus* around 60 eggs and *P. leniusculus* around 110 eggs per year. The difference in the annual reproductive output is more pronounced at $f=1$, resulting in $\approx $40 eggs per year (*A. torrentium*; i.e. 80 eggs per clutch if reproducing every second year; Parvulescu, 2019), $\approx $190 eggs per year (*A. astacus*) and $\approx $320 eggs per year (*P. leniusculus*). In other words, the invasive *P. leniusculus* has at moderate food availability ($f \approx 0.7$) a $\approx $7 times higher annual reproductive output compared with *A. torrentium*, which becomes eight times higher at $f=1$. Annual reproductive output of *P. virginalis* at 12.5$^{\circ }$C by far outcompetes the reproductive output of Astacidae at any compared food availability, with around 300 eggs per year at $f \approx 0.7$ and 700 eggs per year at $f=1$. The predicted annual reproductive output of *P. virginalis* is—due to a suboptimal simulated temperature for this species (see next section)—relatively low in the context of higher field observations, but it is nonetheless, 17 and 20 times higher compared with *A. torrentium* and almost 4 and 5 times higher compared with *A. astacus* , at $f$ of 0.7 and 1, respectively.

***Effects of temperature*** A temperature increase of 1.5 to 4.5 degrees relative to the set baseline temperature of 12.5$^{\circ }$C (at $f=1$) did not affect the predictions for ultimate length and length at puberty, but had a pronounced effect on age at puberty and the reproductive output (Fig. 3 in the main article and Figs B.8 and B.9 in the Appendix).

All species matured faster at higher temperatures, reducing the age at puberty by up to 40% for Astacidae (*A. torrentium*, *A. astacus* and *P. leniusculus*) and by up to 60% for *P. virginalis*: predictions for age at puberty at 12.5$^{\circ }$C range between 664 days (*P. virginalis*) and 1237 days (*A. astacus*), and at 17$^{\circ }$C between 260 days and 690 days (for the same two species). Annual reproductive output of *P. virginalis* again displays the strongest effect, increasing with environmental temperature from 700 eggs per year to 1700 eggs per year (+140%). By contrast, the annual reproductive output of the Astacidae species within this temperature range increases with temperature by $\approx $ 75% in all three species. The simulated temperature increase would therefore result either in more clutches per year, or in larger clutches. For example, for *P. virginalis* at the highest simulated temperature (17$^{\circ }$C) we could observe 5 clutches of 300–400 eggs roughly 70 days apart, or 3 clutches of 500–600 eggs roughly 120 days apart, adding up to 1700 eggs per year.

***Comparing the metabolic response of A. astacus and P. leniusculus*** We compared two direct competitors—the native *A. astacus* and the invasive *P. leniusculus*—by simulating a hypothetical environment. The environment was characterized by a certain food density ($X$) and temperature ($T$), used as forcing variables for *A. astacus* and *P. leniusculus* models. The same absolute food density ($X$) results in a different scaled functional response $f$ for each species, due to differences in metabolism expressed as different values of parameters, notably $\{\dot {p}_{Am}\}$, $\kappa _X$, and $\{\dot {F}_m\}$, which define the half-saturation coefficient of scaled functional response, $K_X$ (Eq. 1). In the simulations, the value of $K_X$ is driven by the estimated value of $\{ \dot {p}_{Am} \}$ (see Table 2), because available data were not sufficient to estimate the other two parameters.

Under all simulated scenarios (see Methods or caption of Fig. 4 for description) the native *A. astacus* will grow to a larger ultimate length compared with *P. leniusculus*, even though the absolute food density is the same for both species. This is a consequence of a higher value of $\{ \dot {p}_{Am} \}$ estimated for *P. leniusculus* (Table 2), which translates the food density to a relatively lower $f$ for *P. leniusculus*. Under ‘control’ conditions (scenario (1): temperature of 12.5$^{\circ }$C and initial $X$), $f=0.8$ for *A. astacus* and $f=0.75$ for *P. leniusculus*, whereas under scenarios with a 20% decrease in food density (scenarios (2) and (3)), $f=0.76$ for *A. astacus* and $f=0.71$ for *P. leniusculus*. Size is an important factor for food competition, but the relative advantage of *A. astacus* is small (2% to 3% difference in ultimate length).

The reproductive output of the invasive *P. leniusculus*, despite the 2–3% smaller ultimate length and lower perceived food availability ($f$), is by 25–30% higher than that of *A. astacus* in all simulated scenarios (Fig. 4). A 2-degree increase in temperature offsets the negative effects of a decrease in environmental food density, identifying a hypothetical situation when *A. astacus* has a similar annual reproductive output compared with *P. leniusculus*: should *only A. astacus* experience a 2-degree increase in environmental temperature, it can annually produce roughly the same (or even slightly higher) number of eggs as its competitor *P. leniusculus* experiencing the same food availability (darker color circles compared with lighter color triangles in Fig. 4). By contrast, should only *P. leniusculus* experience an increase in average environmental temperature, its reproductive output will be roughly double that of *A. astacus* given the same environmental food density.

## 4 Discussion

Besides through disease transmission, invasive crayfish often displace native European crayfish populations through competitive exclusion due to, among others, their broader physiological tolerance and advantageous life history traits (Kouba *et al.*, 2021). Thus, we used DEB models to identify main conservation issues with respect to food availability and expected increases in temperature due to climate change. We do this by comparing the impact of food and temperature on physiological performance of two successful invaders (*P. leniusculus* and *P. virginalis*) and two vulnerable native European species (*A. astacus* and *A. torrentium)*.

### 4.1 Insights on the physiology of crayfish

We created DEB models for two and extended existing DEB models for additional two species of crayfish. The models were parameterized using above-average quality and completeness of data (AmP, 2021: About page), sufficient to independently estimate energy utilization in both the somatic and the reproductive branch. Hence, the models represent ontogeny of target species well. The resulting ensemble of models and parameters enabled us to compare physiological performances of successful invasive and of vulnerable native European crayfish and to examine possible sources of observed metabolic limitations and advantages of a particular species.

Currently, some of the examined species either co-occur or have co-occurred in the wild, in particular (i) *P. leniusculus* with *A. astacus* or A*. torrentium*, (ii) *A. astacus* with *A. torrentium* and (iii) *P. virginalis* with *A. astacus* (Chucholl & Schrimpf, 2016; Ercoli *et al.*, 2014; Grandjean *et al.*, 2021; Rusch *et al.*, 2020; Weinländer & Füreder, 2009; Westman *et al.*, 2002). Most co-occurrences were recorded for the invasive *P. leniusculus* outcompeting the native *A. astacus* (Westman *et al.*, 2002). Given DEB parameters for the two species, this is not surprising: *P. leniusculus* has a higher assimilation rate (Table 2), matures faster and reproduces more at all food levels and temperatures (Figs 2 and 3). Analyses of DEB parameters and simulations suggest that *P. leniusculus* is the superior competitor also compared with the native *A. torrentium* (Figs 2 and 3); similar scenario of *P. leniusculus* dominance is therefore expected as contacts between the invasive *P. leniusculus* and the native *A. torrentium* increase.

In addition to favorable physiological traits evident from the obtained DEB parameters, the much higher aggressiveness of *P. leniusculus* compared with both *A. astacus* and *A. torrentium* along with its larger size compared with *A. torrentium*, enhances its ability to successfully outcompete these vulnerable native species (Söderbäck, 1991, Vorburger & Ribi, 1999). The competitive superiority of *P. leniusculus* in plague-free populations has been frequently observed in the wild (Westman *et al.*, 2002), and developed DEB models offer a mechanistic explanation of underlying metabolic processes.

DEB models also suggest that the emerging invader, *P. virginalis*, has a clear potential to be considerably better competitor than any of the remaining three investigated species. Most importantly, due to the large investment in reproduction compared with growth (lowest $\kappa $), and parthenogenesis, *P. virginalis* is able to reproduce order of magnitude more than all other species (Fig. 2). Furthermore, *P. virginalis* is also their direct competitor: they eat the same food and share the same living space. In such situations, the smaller species is usually at an advantage: it needs less food to survive and is also typically able to utilize more efficiently less abundant (optimal) food sources. This is the case here as well: *P. virginalis* is the smallest species and can reproduce at much lower ($f<0.3$) food availability than other species ($f>0.4$). In line with our model, experimental studies recorded higher growth rates of *P. virginalis* juveniles over *P. leniusculus* (Kouba *et al.*, 2021). They also recorded consistently higher survival rates of *P. virginalis* in competitive trials. This demonstrates that *P. virginalis* could be an effective competitor to the larger-sized *P. leniusculus*, despite the size being one of the major determinants of success in agonistic interactions in crayfish (Hudina *et al.*, 2011a). Temperature dependence only adds to potential of *P. virginalis* for dominance: as temperature rises due to climate change, the competitive edge of *P. virginalis* only increases.

### 4.2 Environmental simulations

Simulations of theoretical environments — each characterized by food availability ($f$ used as a proxy) and temperature ($T$) — enables a direct comparison of growth and reproduction of the four studied species exposed to similar conditions. Analysing selected life history traits (ultimate length, length and age at puberty, annual reproductive output) as a function of $f$ and/or $T$ helps us understand how these traits will change relative to the environmental changes. While food *per se* is not a limiting resource for crayfish, competition for food may prevent subdominant species from accessing it, since (preferred) food may be patchily distributed in the habitat—in high-density populations where the competition intensity is increased and interspecific interactions are more frequent, it is possible to observe disruption in feeding activities and weight loss in individuals (Hudina *et al.*, 2011a).

The simulated scaled food availability ($f$) ranged from the value barely supporting maturation, to the value exceeding the theoretical maximum. Extremes of the range are ecologically less relevant: at low extremes the population would not grow, and values higher than $f=1$ are anomalous, but could explain large size of *P. virginalis* reported by Kouba *et al.* (2021) and Vogt *et al.* (2015). Effects of simulated levels in food availability varied between species, but a general trend can be observed, where the small *P. virginalis* belonging to Cambaridae responds to favorable environments by increasing its ultimate length by a much larger factor (+260%) compared with the three crayfish belonging to the Astacidae family (+114% to +150%).

Simulated changes in food availability show a markedly higher effects on reproduction rate of the new invader *P. virginalis* than the other species. This is a clear consequence of a much higher proportion of energy directed to reproduction (30% for *P. virginalis* vs. 4–7% for the other species). Given the large investment into reproduction, any increase in food availability will dramatically increase the reproductive output of *P. virginalis*, but will only have marginal benefits for the other species. The invasive *P. leniusculus* also has a considerably higher reproduction rate than the two native species, but still lags behind *P. virginalis*. The higher reproduction rates of invasive crayfish reflect current knowledge: successful crayfish invaders are in general competitively superior and exhibit higher fecundity, earlier maturation and broader tolerance to environmental stress (Kouba *et al.*, 2021), and may thus maintain higher reproductive output and earlier maturation even in suboptimal conditions of low food availability due to, e.g., competition in co-occurring populations, as shown in this study.

While increasing food availability decreases time to puberty in all species, *P. virginalis* is special in that it can mature at much lower food levels ($f<0.3$ vs. $f>0.4$ for other species). The difference can be attributed to the phylogenetic and the resulting biological differences between *P. virginalis* (family Cambaridae) and other analysed species (family Astacidae): overall faster life cycle of *P. virginalis* with much shorter life span, earlier maturation, shorter egg incubation period and higher reproduction frequency resulting from parthenogenetic mode of reproduction (Vogt, 2020; Table 3).

Climate change projections predict that global temperatures will continue to rise (IPCC, 2013; Knutti & Sedláček, 2013). This will have grave consequences for freshwater ecosystems and their biota, as the temperature regime changes will affect both water quality and quantity (Capon *et al.*, 2021). As described in the Methods section, we established 12.5$^{\circ }$C as the reference mean annual water temperature representative for all studied species, at least in the Continental biogeographical region of Europe where all studied crayfish occur (Kouba *et al.*, 2014), but also by taking into account general differences between other biogeographical regions of species occurrence (i.e. Mediterranean, Alpine, Pannonian bioregion; Maguire & Gottstein-Matočec, 2004). Simulations show that realistic temperature increases from a baseline of 12.5$^{\circ }$C will not cause thermal stress to any of the examined species. Even under the most extreme scenario (RCP 8.5 applied to the selected baseline), temperature will remain within the limits of optimal thermal ranges of three examined species (native *A. astacus*, *A. torrentium* and the invasive *P. leniusculus*; Jaklič *et al.*, 2014). Even though it reproduces more and grows faster than the other three species, with the optimal range between 18$^{\circ }$C and 26$^{\circ }$C (Vogt, 2020), *P. virginalis* is currently, at 12.5$^{\circ }$C, under temperature stress. Thus, projected temperature increase will only shift it towards its thermal optimum and significantly increase its reproductive output.

To explore the extent to which the choice of 12.5$^{\circ }$C as baseline temperature affected the results, we repeated all of the simulations also with baseline temperature set to 16$^{\circ }$C. The results of these additional simulations are presented in the Appendix (Figs B.10 and B.11) and suggest the effect of baseline temperature choice on the general conclusion is minor. More precisely, the same overall pattern emerges: the invasive species outcompete the native ones in terms of both size and reproductive output, and out of the two invasive species, *P. virginalis* is by far the superior invader, especially in the RCP 8.5 scenario with the highest temperature increase.

**Fig. B.11:**
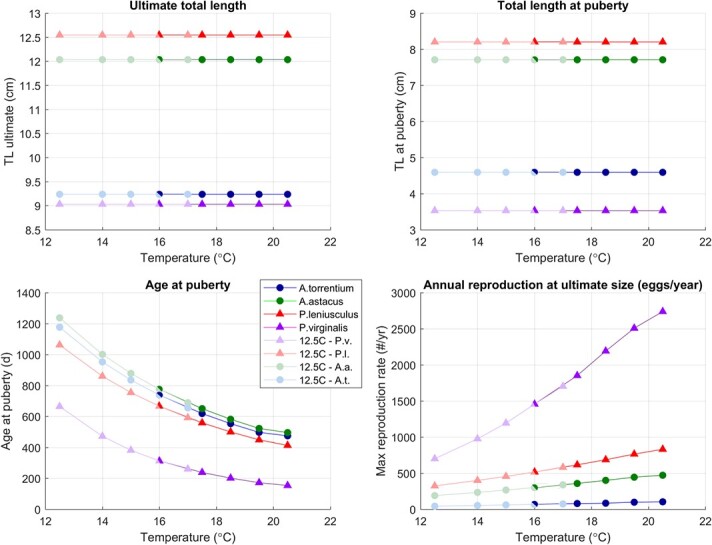
Simulation for current conditions (baseline temperature of 12.5$^{\circ }$C ) and a temperature increase of 1.5 to 4.5 degrees. Different colors and markers denote different species: native species are plotted with circles and invasive species with triangles. Reproduction of all crayfish is expressed on annual basis to enable direct comparison: we used the predicted reproduction of *A. torrentium* (simulated as once every two years, Parvulescu, 2019), and reproduction of *P. virginalis* (simulated as five times per year, Vogt *et al.*, 2004) to calculate the reproductive output per year. Light colors are the simulations at baseline temperature of 12.5$^{\circ }$C (described in the main text), and more intense colors the additional simulations with 16$^{\circ }$C set as baseline temperature.

Expectantly, temperature increase will have no direct effects on either ultimate or at-puberty crayfish size (Fig. 3, also Fig. B.11 in the Appendix), and will even exert positive effects on crayfish maturation and reproduction (earlier maturation, higher reproduction output; Fig. 4, see also Marn *et al.*, 2017a). Again, as with changing food availability, the effects will be most pronounced in phylogenetically distinct *P. virginalis*, whose reproductive output is predicted to steeply increase and maturation age substantially decrease with increasing water temperature. Therefore, climate change will only benefit invasions of *P. virginalis*, which will reach its peak reproductive potential under the most pessimistic scenario (RCP 8.5). Climate change may thus not only increase the suitable geographic range for *P. virginalis*, but may also optimize metabolic processes of this warm-water species. This, in combination with our results, shows that *P. virginalis* should become a more prominent invader in the future.

Despite the strong positive impact of temperature increase on maturation and marginally positive impact on reproductive output of the native *A. astacus*, temperature increase may severely affect its current (limited) distribution range. This has already been observed for *A. astacus* in Croatia, where most of the populations of highest genetic diversity are positioned within areas predicted to become unsuitable for the species under RCP 4.5 and 8.5 scenarios, and may ultimately lead to loss of these populations (Lovrenčić *et al.*, 2022). Interestingly, the same study also indicated substantial decrease of future habitat suitability for *P. leniusculus* (Lovrenčić *et al.*, 2022). *Austropotamobius torrentium*, as a highly vulnerable species with the lowest reproductive output under both optimal food availability and increasing temperature and with a similar trend of loss of suitable habitat (Maguire et al., unpublished data), will be especially endangered in the future climate change and invasive crayfish range expansion scenarios. This calls for specific conservation measures of assisted migration to climate change refugia, spatially isolated from invasive crayfish spread in the case of *A. torrentium*, and for a combination of assisted migration and repopulation measures in the case of *A. astacus* (Lovrenčić *et al.*, 2022).

Comparison between the two physiologically most similar species with high number of recorded overlapping populations — the native *A. astacus* and the invasive *P. leniusculus*—expectedly shows that the invader is the superior competitor. Both species share similar habitat and food preferences, and exhibit synchronous life cycles. Thus, we examined isolated and joint effects of a decreasing food availability and increasing temperature in a simulated case of their co-occurrence. These simulations have demonstrated that under all scenarios, the invasive *P. leniusculus* had an overall higher reproductive output, despite individuals reaching a somewhat smaller size compared with the native *A. astacus* in those particular food density settings. While food availability had a more pronounced effect on the invasive *P. leniusculus* reproduction, its overall reproduction rates remained approximately 1.5 times higher compared the native *A. astacus* in all simulated scenarios (Fig. 4). These findings are congruent with current knowledge and field observations of competitive dominance of *P. leniusculus* over *A. astacus* (Ercoli *et al.*, 2014; Westman *et al.*, 2002) due to, among other factors, its higher aggressiveness and higher fecundity. This study additionally shows that such competitive advantage will remain the same even in the context of climate change or even slightly increase for higher temperatures, thus exerting additional pressure on freshwater ecosystems, as suggested for other freshwater crustaceans (Pellan *et al.*, 2016).

### 4.3 Outlooks and conservation implications

Parameter estimation is constrained by data (un)availability, and parameters that were not well defined by data, should be interpreted and used with caution. For example, even though the small value of maximum assimilation rate ($\{\dot {p}_{Am}\}$) of *A. torrentium* is in accordance with its small ultimate size, the value of the parameter is relatively low compared with other Astacidae (Table 2) and therefore could have been underestimated; more certainty in the parameter value could be gained by obtaining growth curves (in length and/or wet weight). Datasets for Astacidae (*A. torrentium*, *A. astacus*, *P. leniusculus*) were all obtained at one (or more) temperatures within their temperature optimums, so it was impossible to independently estimate the parameters needed to compute $c_T^{(5)}$ temperature correction factor and correct the metabolic rates outside of the temperature optimum. Even though freshwater crayfish most likely share metabolic pathways of enzyme inactivation at critical temperatures, which would justify using the same values for critical high and low Arrhenius temperature parameters (Table 2), it is also possible that the stress response of Astacidae and Cambaridae is drastically different (e.g., Kuklina *et al.*, 2022). This highlights the importance of the impact of pyhlogenetic aspect of species physiology, i.e., that phylogenetically close species are likely to have similar metabolism and ontogeny. This aspect has already been hypothesized in the context of DEB parameter values (Jusup *et al.*, 2017a; Marn *et al.*, 2018) and confirmed with multi-dimensional scaling (Kooijman *et al.*, 2021).

Estimates of other parameters could be improved as well. Weibull aging acceleration ($\dot {h}_a$) that affects the lifespan could benefit from additional survival data, preferably at multiple temperatures. Somatic maintenance ($[\dot {p}_M]$) would benefit from oxygen consumption data. Parameters involved in feeding (digestion efficiency $\kappa _X$ and maximum searching rate $\{\dot {F}_m\}$) were only approximated using default values. Replacing the default values with those estimated using measured data on ingestion would increase accuracy and therefore the value of *A. astacus* and *P. leniusculus* direct comparison, because these parameters, together with the estimated $\{\dot {p}_{Am}\}$, affect the half-saturation constant $K_X$ and the resulting value of the scaled functional response $f$.

Despite the limitations and uncertainties linked to some parameter values of some species, the obtained parameter sets have good predicting power due to the covariation parameter estimation method (Lika *et al.*, 2011; Marques *et al.*, 2019), which simultaneously takes into account all types of data and different underlying processes thus ensuring consistency of the parameter set. Given the phylogenetic aspects of physiology, the well-determined model parameters of the four studied species could also be useful in assessing metabolic traits of other phylogenetically related species for which data gaps may be much larger.

In this study, we applied the baseline temperature, as well as its projected increase, as a constant throughout an individual’s life span, with the aim to identify and analyse general species-specific metabolic differences. In reality, some crayfish, especially the *P. virginalis* that generally prefers warmer waters and has an overall faster and shorter life cycle, would probably spend a part of their lifetime experiencing higher-than-simulated temperatures, for example by selecting specific micro-locations or by targeting the warmer period of the year for maturation. This would speed up the maturation and other metabolic processes. Should seasonal fluctuations or a specific site be of interest, the presented models can include current and historical site-specific field-data (where and if available) as input forcing variables.

In conclusion, our results suggest the alarming and increasing invasion potential of *P. virginalis* in the context of climate change, especially given that Vogt *et al.* (2015) report individuals carrying 720 eggs and females reproduce multiple times per year (Vogt *et al.*, 2004). If aggressive and successful enough, the larger species may deny the smaller one access to food, thus creating a competitive advantage for itself. Indeed, analysis of DEB parameters suggest that such exclusion is the only way any of the three larger species could avoid being supplanted by *P. virginalis*. Considering the new results presented here, we suggest that knowing whether any of the three larger species can successfully physically deny *P. virginalis* access to food may be the key to conservation planning. Current knowledge from staged laboratory trials suggests this is unfortunately not the case: *P. virginalis* has exhibited the ability to outcompete and dominate other successful crayfish invaders (cf. Dobrović *et al.*, 2021). Still, many questions about *P. virginalis* remain, such as whether observed laboratory dominance would translate to dominance in the field given the size difference between the species (see, e.g., Roessink *et al.*, 2022), or whether the observed tolerance to some strains of crayfish plague agent also translates to other strains relevant in natural settings. Thus, further research into this potentially super invader is clearly necessary.

It is our hope that models presented here guide conservation efforts for native species and management strategies for invasive species, fulfilling a much needed role of predictive models in conservation physiology (Cooke *et al.*, 2013; Lavaud *et al.*, 2021; Taylor *et al.*, 2019). We have used the models to simulate responses (growth and reproduction) of the four species under similar constant environmental conditions: a range of temperatures and a range of scaled food availabilities. The simulations can be expanded by adding a broader temperature range and/or by fluctuating the environmental parameters (food availability or temperature, or both). This can help to identify conditions that limit or favor a particular species, and thus help identify actual areas with such characteristics, with the idea of focusing conservation or management activities to a well chosen region within a species habitat.

### Data Availability Statement

The data and code underlying this article are available in the Add-my-Pet library (AmP, 2021) at https://bio.vu.nl/thb/deb/deblab/add_my_pet/entries_web and can be accessed directly by using the species name (*Austropotamobius torrentium* or stone crayfish, *Astacus astacus* or noble crayfish, *Pacifastacus leniusculus* or signal crayfish and *Procambarus virginalis* or marbled crayfish).

## Figures and Tables

**Fig. A.3:**
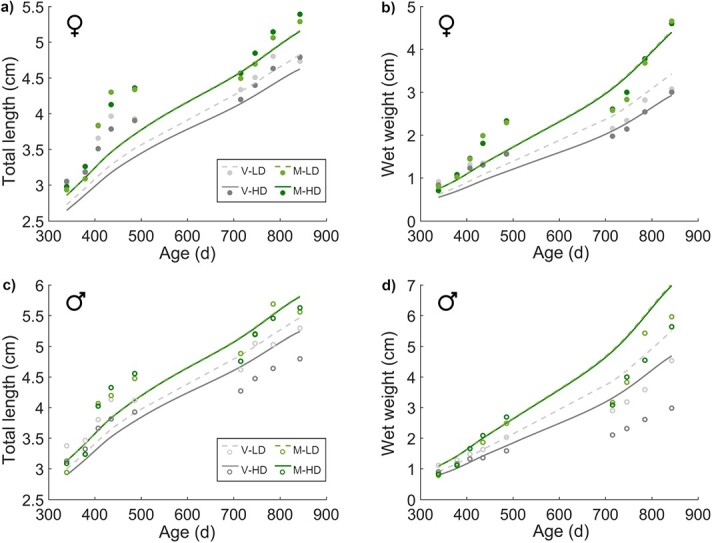
Noble crayfish, *Astacus astacus* - data (markers) and model predictions (lines). Total length and wet weight vs. age for Females (a, b) and Males (c, d) for different food sources and densities. V-LD, M-LD, V-HD and M-HD stand for Vegetable and Meat diet, Low and High Density, respectively. Data taken from Basta (2014). Relevant parameters for model predictions are given in Table 2. Dataset specific scaled functional responses are as follows: V-LD - 0.6402; V-HD - 0.6019; M-LD - 0.7079; M-HD - 0.7061

**Fig. A.5:**
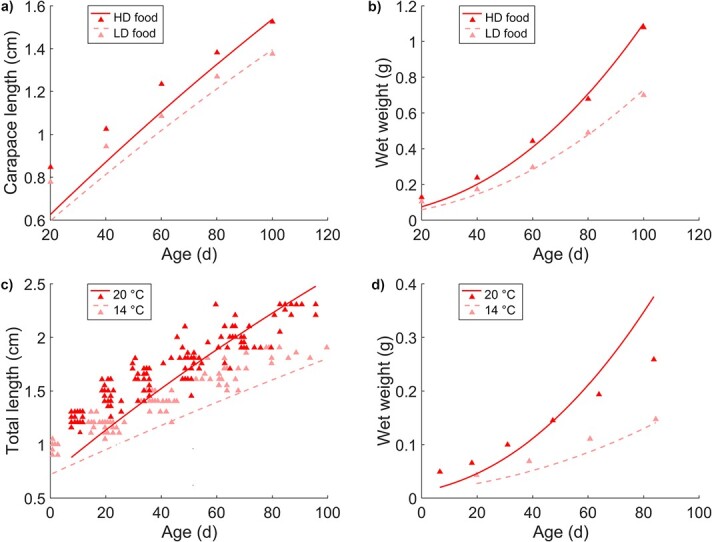
Signal crayfish, *Pacifastacus leniusculus* - data (markers) and model predictions (lines). Length and wet weight vs. age, at different food densities (a, b) and temperatures (c, d). HD and LD food stand for High and Low food Density, respectively. Data sources: (a) and (b) González *et al.* (2010); (c) and (d) Kozák *et al.* (2009). Relevant parameters for model predictions are given in Table 2. Dataset specific scaled functional responses are as follows: González *et al.* (2010) HD food - 0.8780, LD food - 0.7118; Kozák *et al.* (2009) - 0.5750.

**Fig. B.9:**
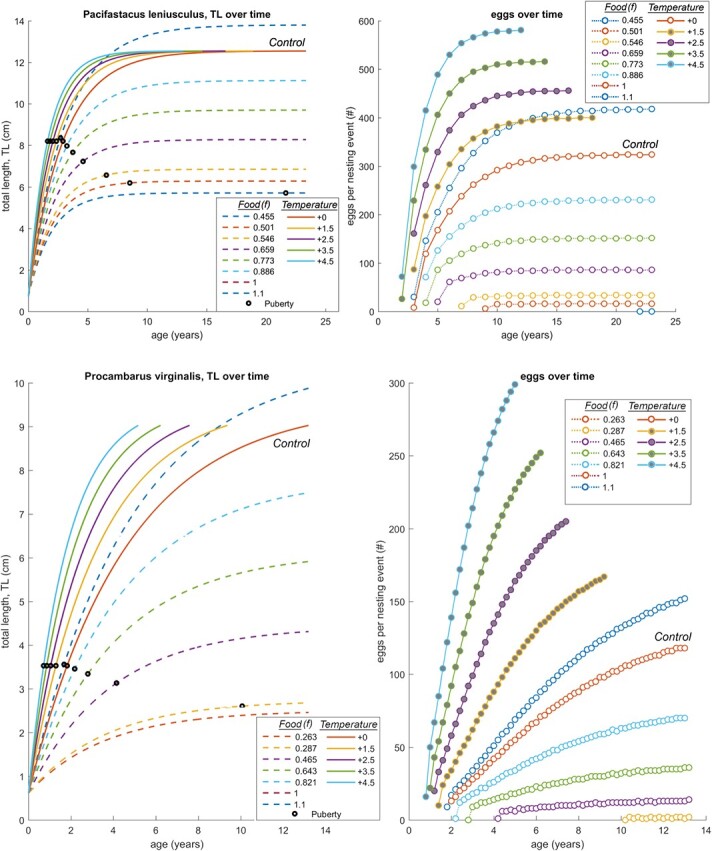
Simulations of different food quality and quantity, and different levels of temperature increase, for the two invasive species: signal crayfish (*Pacifastacus leniusculus*) and marbled crayfish (*Procambarus virginalis*). Reproduction of signal crayfish is annual, and that of marbled crayfish is simulated every 70 days (5 reproductive cycles per year, Vogt *et al.*, 2004). Food simulations are depicted with dashed lines and empty circles, and temperature simulations with solid lines and full circles. For all food simulations, the temperature is 12.C$^{\circ }$C; for all temperature simulations, the food is simulated as $f=1$; the combination of T = 12.C$^{\circ }$C and $f=1$ is marked as ’control’.

## A Appendix: Model parameterization

Models are parameterized (calibrated) separately for each species, using data available in published literature or reports. When laboratory data was available, it was given preference over field data, as food level and temperature were controlled and reported. All data-set or data-point specific comments and supporting information (including $f$-value as the proxy for food availability and temperature) are available online in the mydata_species_name.m and pars_init_species_name.m files in the Add-my-Pet collection (AmP, 2021), where species_name should be replaced with the Latin name of the species, e.g. mydata_Astacus_astacus.m. Here, we list a few assumptions and choices that we made during parameter estimation, which we find relevant for interpreting model parameters and predictions.

## A.1 The model represents an average individual of a species

Values reported for any given property (size or age at a certain life stage) are frequently part of a relatively wide range. We as modellers, however, need to use a single value—as opposed to a range—as model input. The general idea is that we are modelling an average individual of a certain species, and therefore the practice is not to take the largest or the oldest individual ever recorded, or the youngest ever to mature, but the average.

When using a single life history data value for model input, we ignore inter-individual variability. The variability can be reproduced to an extent when sufficient information on main drivers of inter-individual variability is available. The drivers can either be external, such as food or temperature—as explored here—or physiological, e.g. different maintenance costs, assimilation efficiency, reproduction investment, etc. (see, e.g. Marn *et al.*, 2022, in revision). Available data is at this point far short of the requirements for exploring individual variability from physiological sources.

The comparison of the model predictions is meant to be qualitatively informative and biologically relevant. As the models simulate an average individual of the species (not taking individual variability into the account), there is no information on individual variability from which statistical comparisons of individuals within and between species could be derived. Despite the lack of statistical analysis, we believe that the differences and similarities in parameter values are worth noting because they point towards important differences and similarities in physiological responses of the native and invasive species.

## A.2 Age and size at life events

Life events in the context of dynamic energy budget models are listed as—hatching, birth, puberty and ultimate stage (maximum size and life span). Age at hatching in crayfish corresponds to incubation duration, and age at birth to incubation duration plus days until the onset of independent feeding. In nature, incubation duration of astacides includes a cold diapause, sometimes lasting several months (Holdich, 2002). Even though the cold diapause seems to increase survival probability of eggs and juveniles (Policar *et al.*, 2004), it is not necessary for successful incubation (Policar *et al.*, 2004). When incubation data from laboratory experiments was available, such as for *Astacus astacus* and *Pacifastacus leniusculus*, we used this data for model calibration (Table 3) and predicted the incubation duration without the cold-induced diapause. This, however, means that the listed age at hatching (i.e. incubation duration) seems unrealistically small when compared with facts on wild crayfish. The cold-induced diapause can be explicitly included in the model as part of the parameter $t_0$ (Table 2), which currently includes a diapause for *Austropotamobius torrentium*. For the other two astacides, $t_0$ includes only the time required for the mobilization system to develop its full capacity ($t_0$*sensu*; Stubbs *et al.*, 2019).

Puberty is the moment when energy investment is diverted from maturation to reproduction, so in crayfish is assumed to correspond to appearance of glair glands. When this information was not available, we assumed puberty occurred relatively recent prior to spawning. Size at puberty and ultimate size, both in terms of total or carapace length and in terms of weight, includes great variability for all species. For the model parameterization, we had to select a number from the reported range, which would best describe an average individual of that species. For that reason, extremes were avoided and values closer to the center of the range were preferred.

More precisely, for length at puberty of signal crayfish, *P. leniusculus*, we have used the data from Atlas of Crayfish in Europe (Souty-Grosset *et al.*, 2006; and review by Hossain *et al.*, 2018), which states that the signal crayfish reaches maturity between 6–9 cm TL (Souty-Grosset *et al.*, 2006) or 7–9 cm TL (Hossain *et al.*, 2018). We have thus used 8 cm as total length at puberty. In the case of the noble crayfish, *A. astacus*, the data regarding size at maturity are based upon the data from the study by Abrahamsson (1971) and also fall in line with the data reported in Holdich (2002) and Hossain *et al.* (2018). *Procambarus virginalis* has been observed to grow to a larger size than roughly 10 cm total length and 23 g wet weight used for maximum size in this study. For example, Vogt *et al.* (2015) report the largest crayfish (of unknown age) in their laboratory study to be of 10 cm total length and 30 g wet weight, and Kouba *et al.* (2021) say that ‘larger size classes (e.g. up to 12–13 cm) can be found in wild populations...’. However, such large size cannot be obtained during the maximum ever recorded lifespan of 1610 days (Vogt, 2010) given the observed growth rate. Moreover, generally maximum observed size in laboratory stocks and wild populations does not exceed 10 cm total length, with ‘the abundance of such [large] size classes typically low...’ (Kouba *et al.*, 2021, and references therein).

## A.3 Scaled functional response, $f$, as proxy for food availability.

Food availability is a speculative factor when modelling wild populations, which is why we work with scaled functional response ($f$) as food proxy. The value of $f$ is often deduced from available data in the context of other available information on metabolism. Taking it a step further, we simulate a range of $f$, to explore the extent to which food availability affects the individual and population dynamics.

Important point to keep in mind is that the scaled functional response ($f = X/(X+K_X)$) takes into account both food quantity and quality, resulting in a specific value of $X$,as well as the metabolism of the individual, via the half-saturation constant, $K_X$. This effectively means that, while no crayfish will be starving due to detrivory/omnivory, some will have access to more nutritious food than others and some will have access to more of that food (defining the value of $X$), all of this relative to species-specific energy needs of a certain individual and taking into account the energetic cost of searching for or competing for food (jointly defining the value of $K_X$).

Food availability was, therefore, used also as a proxy for density-dependent effects: even though food availability *per se* is not a limiting factor in nature due to crayfish omnivory (Holdich, 2002), in high-density populations competition intensity is increased and interspecific interactions are more frequent, which may result in disruption in feeding activities and weight loss in individuals (Hudina *et al.*, 2011a).

In the particular case of comparing *A. astacus* and *P. leniusculus*, both species of crayfish (signal and noble) have access to the same quantity and quality of food ($X$), but the food fulfils a higher proportion of the theoretical maximum for the noble crayfish, than it does for the (energy more demanding) signal crayfish, resulting in different f values.

## Model fits to data sets

Next, we present fits of model predictions to data sets (uni-variate data) used for model parameterization for each species: *A. torrentium* in Fig. A.1, *A. astacus* in Figs A.2 and A.3, *P. leniusculus* in Figs A.4 and A.5 and *P. virginalis* in Figs A.6 and A.7. Data points on life history traits (zero-variate data) and model predictions are listed in Table 3 in the main text of the manuscript.

## B Appendix: Model simulations: food and temperature

The main text focuses on certain end-points as functions of food and temperature: size and age at puberty and annual reproduction and total length at ultimate size. The growth rate, lifespan and seasonal reproduction rate are also affected by these two environmental variables, as depicted in the next few figures: Fig. B.8 for the two native species and Fig. B.9 for the two invasive species of crayfish. For example, notice a relatively strong (negative) effect of temperature on the life span, implied by shorter simulations. This is especially pronounced for *P. virginalis* (lower two panels in Fig. B.9) where the asymptote is not approached in neither size nor seasonal reproductive output. Also interesting is the synergistic effect of food and temperature increase: the 1.5-degree increase in temperature results in a similar reproductive output as the increase in food availability or quality (scaled functional response $f>1$). A temperature increase will result in faster growth rates but same ultimate size, whereas an increase in food availability or quality will result both in faster growth rates *and* in larger ultimate size (left panels in both figures). This is a result of the model setup, but is also something that we see in nature (Marn *et al.*, 2017a).

We ran all simulations at a constant average temperature, not taking into account seasonal fluctuations. The goal of our research was general: i.e. to examine the metabolic responses of four different species under different temperature scenarios. We have therefore set all of the studied species in a constant and identical environment. We chose 12.5$^{\circ }$C as baseline temperature for all crayfish species, calculated as an average for large European river basins (IKSR, 2013; Maguire & Gottstein-Matočec, 2004), where all four studied species occur (see Methods in main text for more detail). In reality, marbled crayfish would prefer warmer waters. The baseline temperature as set in the simulations is below the 15$^{\circ }$C set as the stress-inducing low temperature for *P. virginalis*, based on work by Seitz *et al.* (2005). To explore the extent to which our choice for the baseline temperature affected the results, we ran the same set of simulations for a baseline temperature of 16$^{\circ }$C and present the results in Figs B.10 and B.11. The results point to the same pattern: the invasive species outcompete the native ones in terms of both size and reproductive output, and out of the two invasive species, the marbled crayfish is by far the superior invader.
